# Two promising *Bacillus*-derived antifungal lipopeptide leads AF_4_ and AF_5_ and their combined effect with fluconazole on the *in vitro Candida glabrata* biofilms

**DOI:** 10.3389/fphar.2024.1334419

**Published:** 2024-04-19

**Authors:** Madduri Madhuri, Shivaprakash M. Rudramurthy, Utpal Roy

**Affiliations:** ^1^ Department of Biological Sciences, BITS Pilani, Sancoale, Goa, India; ^2^ Department of Medical Microbiology, Medical Mycology Division, Postgraduate Institute of Medical Education and Research (PGIMER), Chandigarh, India

**Keywords:** Antifungal lipopeptide, *Bacillus* sp., biofilm inhibition, *Candida glabrata*, CV assay, confocal microscopy, XTT assay

## Abstract

**Introduction:** *Candida* species are endowed with the ability to produce biofilms, which is one of the causes of pathogenicity, as biofilms protect yeasts from antifungal drugs. *Candida glabrata* (*Nakaseomyces glabrata*) is one of the most prevalent pathogenic yeasts in humans and a biofilm producer.

**Methods:** The study was aimed at evaluating the combined effects of two highly promising antifungal biomolecules (AF_4_ and AF_5_) lipopeptide in nature, chromatographically purified to homogeneity from *Bacillus subtilis* (*B. subtilis*) and the standard antifungal fluconazole (at different concentrations) to demonstrate *C. glabrata* biofilm formation inhibition. Biofilm production and inhibition were evaluated by quantification of the biofilm biomass and metabolic activity using crystal violet (CV) staining and XTT reduction assays, respectively. Microscopic techniques such as confocal scanning laser microscopy (CSLM) and scanning electron microscopy (SEM) were employed to visualize biofilm formation and inhibition.

**Results and Discussion:** Compared to untreated and fluconazole-treated biofilms, an enhanced *in vitro* anti-biofilm effect of the antifungal lipopeptides AF_4_/AF_5_ alone and their combinations with fluconazole was established. The lipopeptides AF_4_/AF_5_ alone at 8 and 16 μg/mL exhibited significant biomass and metabolic activity reductions. SEM and CSLM images provided evidence that the lipopeptide exposure results in architectural alterations and a significant reduction of *C. glabrata* biofilms, whereas (2′, 7′-dichlorofluorescin diacetate (DCFDA) and propidium iodide (PI) analyses showed reactive oxygen species (ROS) generation along with membrane permeabilization. The estimation of exopolysaccharides (EPS) in AF_4_/AF_5_-treated biofilms indicated EPS reduction. The combinations of fluconazole (64/128 μg/mL) and AF_4_/AF_5_ lipopeptide (16 μg/mL) were found to significantly disrupt the mature (24 h) biofilms as revealed by CSLM and SEM studies. The CSLM images of biofilms were validated using COMSTAT. The FTIR-analyses indicate the antibiofilm effects of both lipopeptides on 24 h biofilms to support CSLM and SEM observations. The combinations of fluconazole (64/128 μg/mL) and AF_4_/AF_5_ lipopeptide were found to disrupt the mature biofilms; the study also showed that the lipopeptides alone have the potentials to combat *C. glabrata* biofilms. Taken together, it may be suggested that these lipopeptide leads can be optimized to potentially apply on various surfaces to either reduce or nearly eradicate yeast biofilms.

## 1 Introduction

Candidiasis is frequently involved in surface-associated biofilm formation. These networks have multifaceted interactions with the host. Biofilms may be considered as aggregates of microbes enmeshed in an extracellular matrix (ECM) consisting of multifarious polymeric components, forming a complex three-dimensional architecture on biotic and abiotic surfaces. Microbial adhesion to biotic and abiotic surfaces has been found to be triggered by extracellular polymeric substances (EPS) (Karygianni et al., 2020). More EPS production constitutes a matrix that acts as a glue, surrounding and clasping together the biofilm structure (Flemming et al., 2016). Of note, *Candida* infections are commonly associated with biofilms that can form either on mucosal surfaces or on plastic surfaces of indwelling devices as well (Hasan et al., 2009). The matrix mannan-glucan complex in biofilm is conserved across *Candida albicans* and non-*albicans* species, including *C. tropicalis* and *C. glabrata* (Kuhn et al., 2002; Dominguez, 2018). It has been opined that biofilms may act as major virulence determinants, could provide more survival advantages for non-*albican*s *Candida* species and *C. albicans* strains (Pannanusorn et al., 2013; Alves et al., 2020). Most of the non-*albicans Candida* (NCAC) species such as *C. glabrata and C. krusei, C. tropicalis, C. parapsilosis* possesses strain dependent susceptibility or intrinsic resistance to azoles, and the antifungal activity of other antimycotic agents during biofilm formation remains poorly understood (Ben-Ami et al., 2013; Fonseca et al., 2014; Zhang et al., 2014; Wang et al., 2021; Zuo et al., 2021; Daneshnia et al., 2023). *C. glabrata* causes difficult-to-treat infections due to its high inherent antifungal resistance, particularly against azoles, and is endowed with the ability to form biofilms on the surfaces of various biomedical devices (Silva et al., 2012; D’Enfert and Janbon, 2015; Pfaller and Diekema, 2007; Cavalheiro and Teixeira, 2018; Timmermans et al., 2018).

Biofilms formed by a variety of *Candida* spp. tend to vary in morphology and density. The biofilm structures contain a heterogeneous polymeric extracellular matrix, providing a protective encasement for the fungal cells. *Candida* spp., in general, proliferate as adherent biofilms (Nobile et al., 2006; Magill et al., 2014), and the aggregated communities offer resistance to antifungals and host immune responses, rendering them difficult to treat or eradicate (Chandra et al., 2001; Donlan and Costerton, 2002; Douglas, 2003). Biofilms have the potential to modulate host immunity throughout various developmental stages. The components of the extracellular matrix, adhesin proteins and secreted enzymes might play a role in modulating host recognition by masking the cell wall components that might interact with the immune system (Zawrotniak et al., 2017). During mature biofilm formation, extracellular matrix components contribute to resistance to host defences, and with the dispersal of fungal cells, a more virulent phenotype might appear to aggravate the pathogenesis. Cells of *Candida* spp. produce biofilms on artificial medical devices such as vascular catheters in hospitals, which are often associated with mortality rates of approximately 30% (Kumamoto, 2002; Kojic and Darouiche, 2004; Tumbarello et al., 2007).

*C. glabrata has* been reported to rapidly acquire drug resistance to multifarious categories of antifungals (Rodrigues et al., 2014). The high antifungal drug resistance, as escalated by the higher minimum inhibitory concentration (MIC) values for azoles, especially FLC indicates a reduced therapeutic response and recurrent candidiasis that may be endowed by the uncanny ability of these yeasts to produce recalcitrant biofilm (Canuto and Rodero, 2002). The scant arsenal of biofilm-fighting drugs may be expanded to include potential alternative peptide drugs. Since biofilms offer resilience to antifungals, therapeutic options have become rather limited, leading to the surgical removal of the implant material and its subsequent replacement. In this context, lipopeptides are deemed promising, with the potential for synergy with standard antifungals (Biniarz et al., 2017). Previously, several antifungal peptides (AFPs) have demonstrated synergistic activities with standard antifungals, ameliorating the efficacy of antifungal therapies.

In our previous studies, the production, purification, biochemical nature, and functional characterization of promising AF_4_ and AF_5_ lipopeptide homologues were described. These peptides demonstrated broad-spectrum antifungal potency against over a hundred *C. albicans*, *Candida* non-*albicans*, and *Cryptococcus* isolates (Ramachandran et al., 2018; Ramesh et al., 2023a, 2023b). The current investigation was aimed at studying the *in vitro* efficacy of FLC alone at different concentrations and the combinatorial effect of FLC and reversed-phase HPLC-purified antifungal cyclic lipopeptides (AF_4_/AF_5_) from the cell-free supernatant of *Bacillus subtilis* against maturing 24 h-biofilms produced by *C. glabrata* ATCC 2001.

## 2 Materials and methods

### 2.1 Purification of antifungal compounds

The lead biomolecules AF_4_ and AF_5_ lipopeptides, which possess antifungal properties, were purified from *B. subtilis* RLID12.1 (Ramachandran et al., 2018; Ramachandran et al., 2018). The specific composition production media was used to culture the bacteria, and a multistep purification method was employed to isolate the antifungal compounds. The compounds were partially purified from 1,200 mL of cell-free culture supernatant by acid precipitation, organic solvent extraction, followed by silica (230–400 µm mesh size) based adsorption chromatography. Subsequently, further purification was carried out by the semi-preparative scale reversed-phase high-performance liquid chromatography (RP-HPLC) system consisting of a quaternary pump (Agilent) and a variable wavelength detector fitted with a Phenomenex Luna C18 column (10 mm × 250 mm, 5 µm) (Ramachandran et al., 2018; Ramesh et al., 2023, 2024). To assess the anti-biofilm activity of AF_4_ and AF_5_ in combination with the standard antifungal drug fluconazole (FLC) 64, 128, and 256 μg/mL were utilized with AF_4_ or AF_5_ at 8 and 16 μg/mL.

### 2.2 *Candida s*trains/isolates and culture conditions

The strain of *Candida* non-*albicans* species from the American Type Culture Collection (ATCC) *C. glabrata* ATCC 2001, known to exhibit resistance to FLC, was used in the present study. All strains and isolates used in this study were revived from the glycerol stock (maintained at −80°C) by streaking a loopful on a Sabouraud dextrose agar (SDA) agar plate and grown for 48–72 h at 37°C. On Hi-CHROMagar^TM^ *Candida* (Chromagar, Hi-Media, Mumbai), a differential agar medium was used to grow *C. glabrata*. Freshly grown *C. glabrata* colonies that appeared creamy white to mauve-pink were used for the study. All the experiments were performed using RPMI-1640 containing L-glutamine, phenol red, 0.2% glucose, and 0.165 M MOPS (morpholinepropanesulfonic acid) (pH 7.0 ± 0.2) medium without sodium bicarbonate. Along with *C. glabrata* ATCC 2001, *C. glabrata* ATCC 90030 (alternatively MTCC 3019), two clinical isolates *C. glabrata* NCCPF 100028 and 100029 obtained from the National Centre of Collections for Pathogenic Fungi (NCCPF, Chandigarh, India) were used, and *C. albicans* ATCC 24433 was used as a positive control. The MIC, MFC, SMIC_50_ and MBEC_50_ values of the AF_4_/AF_5_ lipopeptides were determined against five strains/isolates.

### 2.3 Antifungal susceptibility testing (AFST)

To evaluate the *in vitro* efficacy of novel antifungal lipopeptides AF_4_ and AF_5_ against planktonic cells of *C. glabrata*, minimum inhibitory and minimum fungicidal concentrations (MICs/MFCs) were determined, and these values were compared with the MICs of FLC. The AFST assays were performed by the broth microdilution method following the M27-A3 instructions provided by CLSI (Clinical and Laboratory Standards Instiute 2017) guidelines. Sessile minimum inhibitory concentration (SMIC_50_) and biofilm eradication concentration (BEC_50_) were determined.

MICs, MFCs, SMIC_50_, and BEC_50_ were determined for *C. glabrata* 2001, *C. glabrata* 90030, and two clinical isolates of *C. glabrata* NCCPF 100028 and 100029. Sessile minimum inhibitory concentration (SMIC_50_) was performed as described previously (McCluskey et al., 2005). For each isolate, 100 μL of cell suspension in RPMI-1640 medium adjusted to 1 × 10^6^ CFU/mL was incubated with 100 μL of RPMI containing serially diluted lipopeptide concentrations ranging from 64 to 0.125 μg/mL. The plates were incubated at 37°C at 75 rpm; the positive control consisted of drug-free wells. After a 60 min adhesion phase, non-attached cells were washed with PBS, and the medium was replaced with 200 μL of fresh RPMI. The plate was incubated further at 37°C for 48 h, and an XTT reduction assay was performed as described below. SMIC_50_ is the ability of an antifungal concentration leading to a 50% reduction in biofilm formation compared to a drug-free control (McCluskey et al., 2005). A minimum biofilm eradication concentration (MBEC) is defined as the lowest concentration of the compound capable of eradicating a pre-existing biofilm where the antifungal compound penetrates into the mature biofilm and eradicates it by disturbing the matrix. The determination of MBEC_50_ was performed as described previously (Melo et al., 2011). The determination of the MBECs of all three compounds was performed using sterile 96-well polystyrene flat-bottom plates. Biofilms were produced as described below, and XTT assays were used to determine the eradication of pre-formed biofilms as compared with the growth (drug-free) controls (Melo et al., 2011).

### 2.4 Biofilm formation

Biofilm formations for biomass and metabolic activity reduction assays were assessed according to the methodology described elsewhere (Silva et al., 2009), with a few modifications. Biofilm biomass and metabolic activity of biofilms were determined for *C. glabrata* 2001, *C. glabrata* 90030, and two clinical isolates, NCCPF 100028 and 100029. The *in vitro* biofilm formation assay was carried out by using 96-well flat-bottomed microtiter plates (Melo et al., 2011). For biofilm formation assessment, in order to grow the *Candida* cells*,* a few individual colonies from 24 h sub-cultured plates were inoculated into 10 mL of Sabouraud dextrose broth (SDB) and incubated for 18–20 h at 37°C under agitation (120 rpm). To prepare the inoculum for biofilm formation, cells were harvested by centrifugation at 3,000 *g* for 10 min at 4°C and washed twice with sterile phosphate buffer saline (PBS). A volume of 200 µL of the adjusted yeast cell suspension (10^6^ cells/mL) in RPMI 1640 was added to 96-well polystyrene plates (Pierce et al., 2008). The suspension of *Candida* cells was incubated at 37°C at 75 rpm for 6 h and 24 h for biofilm formation. After incubation, the RPMI medium was discarded, and non-attached cells were washed with phosphate buffered saline (PBS). Next, the antifungal compounds (AF_4_ or AF_5_) at concentrations of 8 or 16 μg/mL were added with fresh RPMI medium to treat the biofilms. In addition, fluconazole (FLC) alone was used as a comparison at varying concentrations of 32, 64, 128, and 256 μg/mL to assess the enhanced efficacy of fluconazole at the same concentrations with lipopeptides (AF_4_ or AF_5_). The purified antifungal lipopeptides were dissolved in sterile 10 mM phosphate buffer, whereas fluconazole at varying concentrations was prepared in Dimethyl sulfoxide (DMSO) for use in the experiments. Wells without drugs served as growth controls, and growth medium without cells served as media controls. Post-treatment plates were incubated for another 24 h for biofilm quantification or morphological analysis.

### 2.5 Crystal violet (CV) staining

To quantify the total biomass of treated biofilms, the CV staining method was utilized as described previously with a few modifications (Silva et al., 2009). Drugs, whether FLC or AF_4_/AF_5_ at different concentrations were added to 6 h or 24 h *Candida* biofilms. In control (untreated), no drug was added. After the addition of antifungals, the plates were incubated for 24 h as mentioned previously. Briefly, the *Candida* biofilms were washed with 200 µL of PBS buffer and fixed with 100 µL of 99% methanol, which was subsequently removed after 15 min. The microtiter plates containing the fixed biofilms were left to dry at room temperature for 5 min, and 200 µL of 1% (v/v) CV in 25% methanol were then pipetted into each well. The excess CV was removed after 20 min of incubation and the wells were washed with autoclaved distilled water, allowing the bound CV to be released by adding 250 µL of 33% acetic acid (v/v) in water. The absorbance values were obtained at 590 nm (Melo et al., 2011). The blanks were considered wells containing RPMI 1640 supplemented with 0.2% glucose without fungal cells. For FLC treatments, dimethyl sulfoxide (DMSO) was used alone in the wells as a control (untreated).

### 2.6 XTT [2,3-bis-(2-methoxy-4nitro-5-sulfophenyl)-2H-tetrazolium-5 carboxanilide] colorimetric reduction assay

The XTT reduction assay was used to quantify the metabolically active cells after treating them with a single drug or combination of drugs. In principle, XTT gets reduced by the respiratory chain enzymes present in the cell membrane, whereas tetrazolium salts are reduced by mitochondrial dehydrogenases in yeast cells (McCluskey et al., 2005). The XTT reduction assay was used to quantify biofilm metabolic activity as a measure of biofilm production. Since metabolically active cells reduce this compound to water-soluble formazan, this reduction assay only counts viable cells. Briefly, the process of biofilm formation was performed according to the method prescribed above. The biofilm-inhibitory effect was evaluated by calculating the percentage reduction in biofilm growth compared to untreated controls. To assess the inhibitory effect on pre-formed biofilm during micro-colony formation, referred to as the developmental-phase (6 h) biofilm, AF_4_/FLC and AF_5_/FLC were added after 6 h. For the maturing stage biofilms, the drug components were added at 24 h and incubated for another 24 h. Post-treatment, metabolically active cells in the biofilm were evaluated by adding 200 μL XTT solution containing menadione to the reaction, which was then incubated in the dark for 3 h at 37°C. Readings were taken at 492 nm by a microplate reader (Ramage et al., 2001). The percentage of biofilm reduction was calculated with the appropriate equation. For FLC treatments only, DMSO at the same concentration in the wells was used in the controls.

### 2.7 Field emission-scanning electron microscopy (FE-SEM)

The morphological and architectural alterations of treated biofilms may be observed and analyzed by FE-SEM. Biofilms were grown on coverslips placed in 24-well culture plates. Prior to use, coverslips were sterilized by autoclaving at 121°C for 20 min, rinsed with 70% ethanol, and dried in a flow chamber with UV light for 15 min. From the 24 h post-treated biofilm wells, the medium was removed, and the wells were washed twice with 350 μL of sterile PBS. Next, the biofilm samples were fixed with 2.5% glutaraldehyde (60 min at room temperature), washed twice with 0.1 M sodium cacodylate buffer, and then incubated with osmium tetroxide (OSO_4_) for 30 min, followed by a wash with 0.1 M sodium cacodylate buffer. The dehydration process was performed with a gradual dilution of ethanol (30, 40, 50, 60, 70, 80, 90, and 100% for 10 min each). Then, the samples were fixed on aluminium stubs, sputter coated with gold, and observed using a scanning electron microscope (FEI, Quanta 250 FEG 30 kV), using different magnifications.

### 2.8 Confocal scanning laser microscopy (CSLM)

Biofilm was grown on a sterile round-shaped cover slip placed in a 24-well, sterile polystyrene flat-bottom plate. A suspension of *Candida* cells (1 × 10^6^ cells/mL) was incubated at 37°C for 24 h for mature biofilm, and after incubation, non-adherent cells were removed from plates by washing twice with PBS. Further, biofilms were incubated for 24 h in the presence of respective antifungals at different concentrations of AF_4_ 16 μg/mL with FLC 64/128 μg/mL and AF_5_ 16 μg/mL with FLC 64/128 μg/mL combinations compared with FLC 64 and 128 μg/mL treatments alone and control (untreated). Briefly, post-treated biofilms were washed twice with PBS and incubated in the presence of 400 µL of PBS containing the fluorescent stain FUN-1 (1 mL from 10 mM/L stock; Thermofisher) and concanavalin A–Alexa Fluor 488 conjugate 15 µL from 5 mg/mL stock; Invitrogen) for 45 min at 37°C. FUN-1 gets converted by metabolically active cells to orange-red or yellow-orange fluorescent intravacuolar compounds, and the concanavalin A–Alexa Fluor 488 conjugate preferentially binds to α-mannopyranosyl and α-glucopyranosyl residues present in cell wall polysaccharides emitting green fluorescence (Chandra et al., 2001). Images were captured using an LSM710 inverted confocal laser-scanning microscope (Olympus FV3000, CSIF BITS Goa) and analyzed using CSLM Z-Stack analysis: depth measurements were taken at regular intervals across the biofilm, and three-dimensional images of mature biofilms were captured. The examination of important parameters such as maximum thickness, roughness coefficients, and biomass of control and treated images was evaluated using the statistical tool COMSTAT 2.1. After capturing the *Z*-stack images, the biomass (µm^3^/µm^2^), average thickness (µm), and roughness of the biofilm were analyzed.

### 2.9 Determination of intracellular reactive oxygen species (ROS) generation

ROS generation was assayed using the fluorescent probe DCFDA (2′, 7′-dichlorofluorescin diacetate) staining (Gupta et al., 2021). In brief, pre-treated *C. glabrata* biofilm with AF_4_/AF_5_ at 8 and 16 μg/mL was washed thrice using PBS. The fluorescent probe was added at a final concentration of 10 μM, and the cells were incubated at 37°C for 30 min in the dark. The cells were then collected and washed with PBS before the fluorescence intensity was measured at an excitation wavelength of 488 nm and an emission wavelength of 525 nm using a fluorescent spectrometer. The permeability of damaged cells caused by ROS accumulation was measured using a CSLM at 617 nm emission and 543 nm excitation spectra. Representative images of fields in control and treated samples were captured. The permeability of damaged *Candida* cells to propidium iodide (1 mg/ml) caused by ROS accumulation was observed by CLSM at 617 nm emission and excitation at 543 nm wavelengths. Representative images of fields in control and treated samples were captured.

### 2.10 Attenuated total reflectance-Fourier transform infrared (ATR-FTIR) spectroscopy

Determination of the biochemical compositions of treated and untreated *C. glabrata* 2001 biofilms were analysed by ATR-FTIR as previously described (Nithyanand et al., 2015; Pebotuwa et al., 2020) with few modifications. Briefly, 24 h grown biofilms were treated with AF_4_ (8 and 16 μg/mL) or AF_5_ (8 and 16 μg/mL) or FLC (32/64 μg/mL) for 24 h at 37°C, the contents in all wells of the microtiter plates were discarded. The wells were then rinsed twice using sterile distilled water. The biofilm fractions were then scraped from the walls of the wells by pipetting using 0.9% NaCl. The suspensions in the microtiter plate wells were transferred into microfuge tubes and vortexed for 3 min. The tubes were centrifuged at 3,000 *g* for 15 min at 4°C. The resulting cell pellets from treated and untreated biofilms were placed in direct contact with the diamond crystal in the Perkin Elmer Spectrum two FTIR spectrometer ATR-FTIR (USA). The biofilm analyses were performed in the wavenumber range between 3,000 cm^−1^ and 500 cm^−1^ at a resolution of 4 cm^−1^. Each final spectrum was the average of 64 scans. A total of triplicate infrared (IR) spectra were acquired from each of the samples to generate a spectrum of the biochemical composition of *C. glabrata* 2001 biofilm treated with antifungal lipopeptides (AF_4_/AF_5_), FLC, and control (untreated).

### 2.11 Quantification of exopolysaccharides (EPS)

The method described in (Nithyanand et al., 2015), which was used to measure the extracellular polysaccharides in both untreated and treated samples of *C. glabrata* biofilm, was used. Briefly, mature biofilms were grown in a 24-well plate for 24 h, then the drug was added at various concentrations to the respective wells. After 24 h incubation with the respective drug, cells were aspirated by washing with 0.9% saline and transferred to sterile test tubes, and an equal volume of 5% phenol and five volumes of concentrated sulfuric acid were added to the cell suspension. This was followed by dark incubation for 60 min, and the absorbance was recorded at 490 nm (Nithya et al., 2011; Nithyanand et al., 2015).

### 2.12 Statistical analysis

The experiments were performed in triplicates, and results were represented as mean ± standard deviation. Statistical differences among the groups of data were analyzed by one-way ANOVA with Sidak’s *post hoc* test. In all the comparisons, a *p*-value of 0.05 or lower was considered significant. The analyses were done in the software Graph pad prism Software version 9.3.1.

## 3 Results

### 3.1 Effect of novel antifungal lipopeptides AF_4_/FLC and AF_5_/FLC on the preformed biofilm

The antifungal combination therapy was aimed at testing antibiofilm efficacy by developing a potential combination (Ashley and Johnson, 2011). The AF_4_ and AF_5_ were the reverse-HPLC-purified fractions (Supplementary Figures S1A, B) that demonstrated broad-spectrum antifungal activities against over one hundred fungal isolates/strains (Ramachandran et al., 2018).

The MIC and MFC values of AF_4_ and AF_5_ against *C. glabrata* ATCC 2001 were 4 μg/mL for each compound, and the AF_5_ lipopeptide exhibited MFC at 8 μg/mL. Interestingly the MIC and MFC values of the AF_4_/AF_5_ against *C. albicans* ATCC 24433 were comparable. In our previous study, *C. glabrata* ATCC 2001 cells exposed separately to AF_4_ and AF_5_ lipopeptides demonstrated significant increase in cell membrane permeability and damages revealed by PI-based flow cytometry and FUN-1 based confocal microscopy (Madhuri et al., 2024). The developmental and maturing biofilms enable the *Candida* cells to overcome the effects of azoles and exhibit higher drug resistance. Since simple azole mono-therapy rarely eradicates or disrupts resistant *Candida* biofilms, removal of the infected device becomes necessary for curing the biofilm-associated infections (Pappas et al., 2009). To test biofilm inhibition, 8 μg/mL (2× MIC) and 16 μg/mL (4× MIC) concentrations of AF_4_/AF_5_ lipopeptides were used, while fluconazole (FLC) was used at 2×, 4×, and 8× MICs. The lipopeptide AF_4_ demonstrated an SMIC_50_ value of 8 μg/mL against pre-formed *C. glabrata* biofilms, while the SMIC_50_ value for AF_5_ was double the MFC value, i.e., 16 μg/mL. The results of the MIC, MFC, SMIC_50_, and BEC_50_ values for AF_4_, AF_5_, and FLC against all five strains/isolates have been summarized in (Table 1).

**TABLE 1:** Summary of MICs, MFCs, sessile minimum inhibitory concentrations (SMIC_50_), and biofilm eradication concentrations for AF_4_/AF_5_ tested against *C. glabrata* ATCC 2001*, C. glabrata* ATCC 90030 (MTCC 3019), *C. glabrata* NCCPF 100029*,* and *C. glabrata* NCCPF 100028*,* and *C. albicans* ATCC 24433 (positive control).

No	Strain	Drug	MIC (µg/mL)	MFC (µg/mL)	SMIC 50 (µg/mL)	BEC 50 (µg/mL)
1	*Candida albicans* ATCC 24433	AF_4_	4.0	4.0	8.0	16–32
		AF_5_	4.0	8.0	16.0	64–128
		FLC	2.0	ND	≥512	>512
2	*Candida glabrata* ATCC 2001	AF_4_	4.0	4.0	16–32	32.0
		AF_5_	4.0	8.0	32–64	≥64
		FLC	16–32	≤64	≥256	≥256
3	*C. glabrata* ATCC 90030	AF_4_	4.0	4.0	8.0	8.0
		AF_5_	8.0	8.0	8–16	16.0
		FLC	4.0	16–32	≥256	≥256
4	*C. glabrata* 100028	AF_4_	4.0	4.0	8–16	16.0
		AF_5_	8.0	8.0	8.0	16.0
		FLC	4.0	≥256	≥256	>512
5	*C. glabrata* 100029	AF_4_	4.0	4.0	8–16	8–16
		AF_5_	8.0	8.0	16.0	32.0
		FLC	4.0	≥512	>512	>512

### 3.2 Effect of antifungal lipopeptides on biofilm reduction with crystal violet staining

**FIGURE 1:**
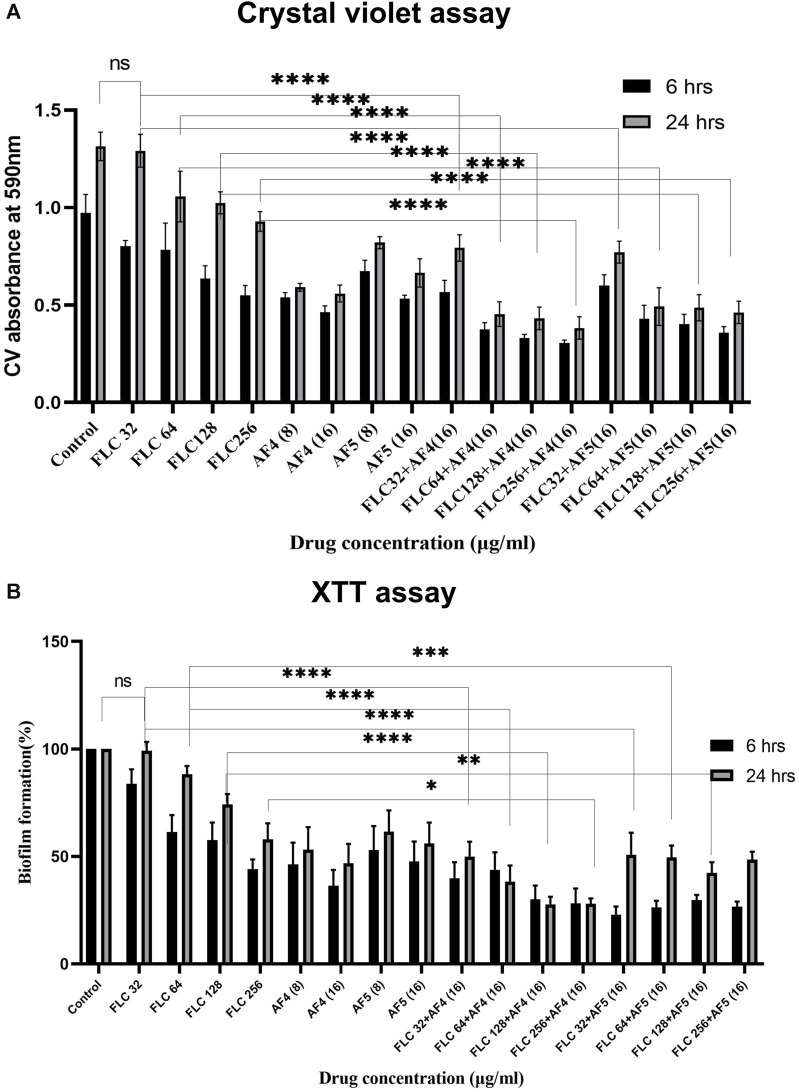
**(A)** Mean absorbance values at 590 nm obtained from the total biomass quantification by CV assay of *CG* 2001 biofilm. The solid rectangular black and gray bars indicate 6 and 24 h biofilms respectively. **(B)** Effect of FLC and FLC plus (AF_4_/AF_5_) on *CG* ATCC 2001 biofilm formation by XTT assay. Graph shows (black bars) 6 h, (white bars) 24 h biofilm. Results were normalized to control (untreated), which was taken as (100%). Data represent the means ± SD of three individually performed experiments with the error bars.

The *in vitro* activity of novel antifungal lipopeptides (AF_4_/AF_5_) in combination with FLC (32–256 μg/mL) against *C. glabrata* 2001 biofilm was determined by quantifying biofilm-forming cell biomass with CV assays at 590 nm (Figure 1A; Supplementary Figures S2A). In comparison to the *C. glabrata* 2001 control, the concentrations of FLC (32, 64, 128, and 256 μg/mL) showed varying levels of biofilm formation inhibition (17.15%, 19.3%, 34.5%, and 43.4%, respectively) for the 6 h developmental-stage biofilm. Whereas, combinatorial studies of AF_4_ or AF_5_ with FLC at various concentrations revealed (63.09%–68.41%) biofilm biomass formation inhibition as compared to control. As far as the maturation phase (24 h) of the biofilm is concerned, FLC alone at concentrations ranging from 32 to 128 μg/mL did not reduce the biofilm biomass produced by *C. glabrata* ATCC 90030, and merely led to a 1.06% reduction in *C. glabrata* 2001 compared to the control (untreated) (Supplementary Figures S2A). When tested on biofilms produced by clinical isolates, a high concentration of 128 μg/mL of FLC resulted in only a (23%) biofilm biomass reduction in *C. glabrata* 100028, whereas a reduction of (48%) was noted in the biofilm biomass of *C. glabrata* 100029 (Supplementary Figures S2A).

Combinatorial studies of AF_4_ or AF_5_ with FLC at different concentrations revealed a significant effect on the 24-h matured biofilm when compared to FLC alone at the respective concentrations. However, the combination of FLC (32 μg/mL) with AF_4_ and AF_5_ appeared to be antagonistic resulting in a biofilm reduction of approximately 43.1% and 41.2%, respectively. In contrast, a significant (*p <* 0.05) biomass (50%–57.4%) reduction by antifungal lipopeptides AF_4_ (8/16) µg/mL and AF_5_ (16 μg/mL) alone as compared to FLC 32 μg/mL alone was observed in the case of *C. glabrata* 2001 (Figure 1A). For another biofilm-forming strain, *C. glabrata* 90030, the lower concentrations of AF_4_ and AF_5_ (8 μg/mL) alone exhibited biomass reductions of 66% and 43%, respectively. For the clinical isolates, at the lower concentration (8 μg/mL), AF_4_ and AF_5_ showed a range of (36%–50%) reduction in *C. glabrata* 100028 biofilm, and (41%–44%) reduction in *C. glabrata* 100029 biofilm, respectively (Supplementary Figures S2A). The combination of FLC with AF_4_/AF_5_ resulted in a rather significant reduction in biomass compared to FLC alone at the same concentration. Of note, a significant (*p <* 0.05) reduction in biomass (65.4% for AF_4_ and 62.5% for AF_5_) was observed when FLC was combined with AF_4_/AF_5_, whereas using the same FLC concentration (64 μg/mL) alone resulted in an insignificant biofilm reduction. Furthermore, combining FLC at 128 μg/mL with AF_4_ and AF_5_ separately resulted in significant biomass reductions of 67.15% and 62.9%, respectively, whereas the treatment by FLC alone at the same concentration resulted in a 20% reduction in *C. glabrata* 2001 cell biomass (Figure 1A).

Similarly, the combinatorial effect of FLC and AF_5_ on *C. glabrata* 90030 biofilm was evident with a reduction of (36%–66%) with FLC (32–128 μg/mL) combined with AF_5_; however, an improved biofilm reduction (61%) was achieved with AF_4_ combined with the lowest FLC 32 μg/mL concentration used in this study. Interestingly, no biofilm reduction (Supplementary Figures S2A) was observed with FLC (32 μg/mL) against the *C. glabrata* 90030 biofilm. Based on the CV-assay results conducted on clinical *C. glabrata* strains, it may be deemed that the lipopeptides AF_4_ and AF_5_ demonstrated more effectiveness in reducing the biofilms compared to their combination with FLC.

### 3.3 Quantification of *C. glabrata* biofilm formation by XTT reduction assay

**FIGURE 2:**
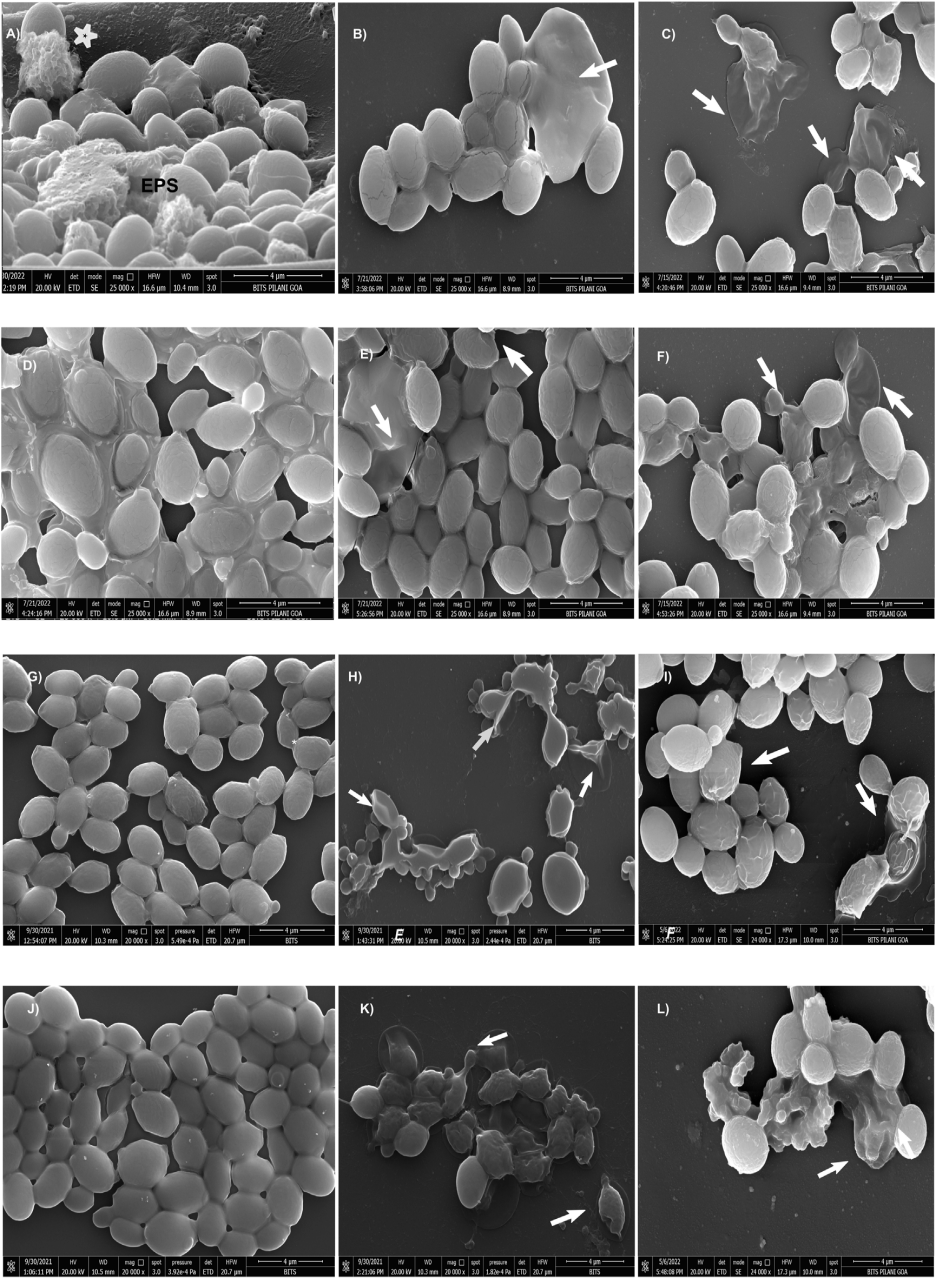
Scanning electron micrographs of *CG* 2001 24 h preformed biofilm. **(A)** control (untreated) and **(B,C)** AF_4_ 8 μg/mL and 16 μg/mL treatments alone respectively, **(D)** treated with FLC 32 μg/mL and **(E,F)** AF_5_ 8 μg/mL and 16 µg/mL-treatments alone respectively. **(G,J)** treated with FLC 64 μg/mL and FLC 128 μg/mL alone respectively, **(H)** treated with FLC 64 μg/mL/AF_4_ (16 μg/mL), **(K)** treated with FLC 128 μg/mL/AF_4_ (16 μg/mL) and **(I)** treated FLC 64 μg/mL/AF_5_ (16 μg/mL), **(L)** treated with FLC 128 μg/mL/AF_5_ (16 μg/mL). Images are shown at 4 µm scale bars. Arrows indicate the cell surface damages and deformities.

The study investigated the efficacy of AF_4_ and AF_5_, individually or combined with FLC, in inhibiting the growth of *C. glabrata* biofilm at different stages. The kinetics of biofilm formation over 6 and 24-h biofilms are illustrated in (Figure 1B). The results demonstrated that the consistent effect of FLC alone on developmental stages could not be consistently observed for the maturing phase (24 h) biofilm. Compared to the *C. glabrata* 2001 control, FLC concentrations (32–256 μg/mL) resulted in a (16.65%–56.27%) reduction in metabolic activity. However, the metabolic activity of biofilms was reduced by (57.4%–74.3%) for AF_5_ and FLC, and (61.4%–77.12%) for AF_4_ (16 μg/mL) and FLC combinations. The results showed that the lowest metabolic activity in the 6 h developmental biofilm was affected by FLC 256 μg/mL in combination with AF_4_ (16 μg/mL), which correlates with the biomass inhibition of the developmental-stage (6 h) biofilm (Figure 1B). No significant difference was observed for FLC-treated 24 h maturing biofilms when compared with untreated biofilms. Only (0.7%–42.6%) biofilm reductions were recorded for FLC when used alone at (32–256 μg/mL) (Figure 2). Interestingly, the significant (*p <* 0.05) reduction of the biofilm activity was observed in the percentage of metabolic activity with the combinations of FLC (64 μg/mL), (128 μg/mL) and (256 μg/mL) with AF_4_ or AF_5_ (16 μg/mL) in *C. glabrata* 2001 (Figure 1B).

The present study revealed that the effective concentration of FLC that reduced the *C. glabrata* 90030 biofilm by 59% was BEC_50_ 256 μg/mL. Besides, AF_4_, when used alone, at a lower concentration (8 μg/mL) demonstrated a BEC_50_ of 54%. A further reduction (62%) was observed when the lipopeptide concentration was increased to 16 μg/mL. Similarly, AF_5_ at 16 μg/mL showed a considerable reduction (65%) in biofilm formation in the case of *C. glabrata* 90030 (Supplementary Figures S2B; Table 1). However, when tested on clinical isolates *C. glabrata* 100028 and *C. glabrata* 100029, FLC at the same concentration of 256 μg/mL could not exhibit a biofilm reduction greater than 23% (Supplementary Figures S2B). On the clinical isolate *C. glabrata* 100028, BEC_50_ was achieved at 16 μg/mL for both AF_4_ and AF_5_. However, in the case of *C. glabrata* 100029, the BEC_50_ values were 16 μg/mL for AF_4_ and 32 μg/mL for AF_5,_ although in either case the FLC BEC_50_ was 512 μg/mL (Table 1). Since biofilm formation is implicated in escalating drug-resistance, these findings underscore the anti-biofilm potential of AF_4_/AF_5_ as these two lipopeptides at low concentrations were able to reduce the metabolic activities of biofilms to an extent that indicates the near-elimination of biofilms. Moreover, CV and XTT results indicate that there is no significant difference between the reductions of biomass and metabolic activity between the treatments of FLC 128 and 256 μg/mL with AF_4_/AF_5_. Therefore, for microscopy, FLC treatments at 256 μg/mL were not considered further.

### 3.4 SEM analysis of *C. glabrata* biofilm

A comprehensive understanding of the biofilm architecture is required for the development of targeted therapeutic approach that aim to treat both preformed biofilm and as well as preventing the formation biofilm. To gain further insight into the effects of AF_4_/AF_5_ alone and the AF_4_/AF_5_ and FLC combination on biofilm architecture and morphology, SEM was performed to visualize 24 h control (untreated) biofilms along with treated biofilms (Figure 2). The SEM images revealed a relatively dense network of yeast cells with tightly packed structures as was observed in the *C. glabrata* 24 h biofilm (Figure 2A). The cells showed healthy, oval-shaped morphological features (Supplementary Figure S3). The formation of a very dense biofilm giving a mat-like appearance at 24 h by *C. glabrata* has been clearly evident from the SEM images (Supplementary Figure S4B) and EPS formation (Figure 2A).

In order to visualize their influence on the development or disruption of *Candida* biofilms, investigations using SEM studies on *in vitro* biofilms treated with FLC (32/64/128 μg/mL) and AF_4_/AF_5_ (16 μg/mL) were carried out. The SEM images of FLC-treated biofilm cells at the concentrations of (32, 64, and 128 μg/mL) appeared as clusters, as illustrated in (Figures 2D, G, J) respectively. Since *C. glabrata* 2001 showed higher resistance to FLC in biofilms, when used alone (32, 64, and 128 μg/mL), FLC did not demonstrate any convincing effect on 24 h biofilm cell morphology, and biofilm cells appeared healthy and oval-shaped in the extracellular matrix. FLC 32 μg/mL-treated-24 h biofilms showed a large number of aggregates and layers of compact cells embedded in the biofilms (Figure 2D) and distinct EPS formation in the biofilms (Supplementary Figure S6A). Even at 64 and 128 μg/mL, FLC-treated biofilms showed clear evidence of biofilms (Figures 2G, J) (Supplementary Figures S6A, B), whereas in contrast to this observation, AF_4_/AF_5_ (16 μg/mL) and FLC 64/128 μg/mL combinations showed not only a considerable reduction in cell number and apparent clearance of biofilm but also discernible cell damages and deformities (Figures 2H, I, K, L) (Supplementary Figures S6C–F). Lipopeptides AF_4_/AF_5_ alone at 8/16 μg/mL proved their *in vitro* anti-biofilm efficacies, as micrographs (Figures 2B, C, E, F) revealed fewer yeast cells in small aggregates lying scattered in biofilms. The ultrastructure alterations induced by AF_4_/AF_5_ (16 μg/mL) alone have been presented in images (Figures 2C, F). Interestingly, significant damages to the biofilm structure occurred with the AF_4_/AF_5_ (16 μg/mL) and FLC (64/128 μg/mL) combinatorial activities, as evident from scanning electron micrographs (Figure 2H, I, K, L). Apparent damages on clusters of biofilm cells were evident from AF_4_ (16 μg/mL) plus FLC 64 µg/mL-treated biofilm (Figure 2H). The combinatorial activity of AF_4_ (16 μg/mL) and FLC at 128 μg/mL on treated biofilms resulted in a loss of structural integrity, though shrinkage of cells was rare in yeast cell morphology, and damages on the biofilm cell wall were evident (Figure 2K). The SEM images (Figures 2I, L) revealed that when treated with a combination of AF_5_/FLC, either at 64 or 128 μg/mL, the morphology of the cells in biofilms was not smooth and healthy compared to only FLC treated biofilms and untreated biofilms. The cell damage and ruptured surface of the biofilm were observed.

### 3.5 Confocal scanning microscope analysis of individual and combinatorial effects of antifungal compounds on *C. glabrata* biofilm

**FIGURE 3:**
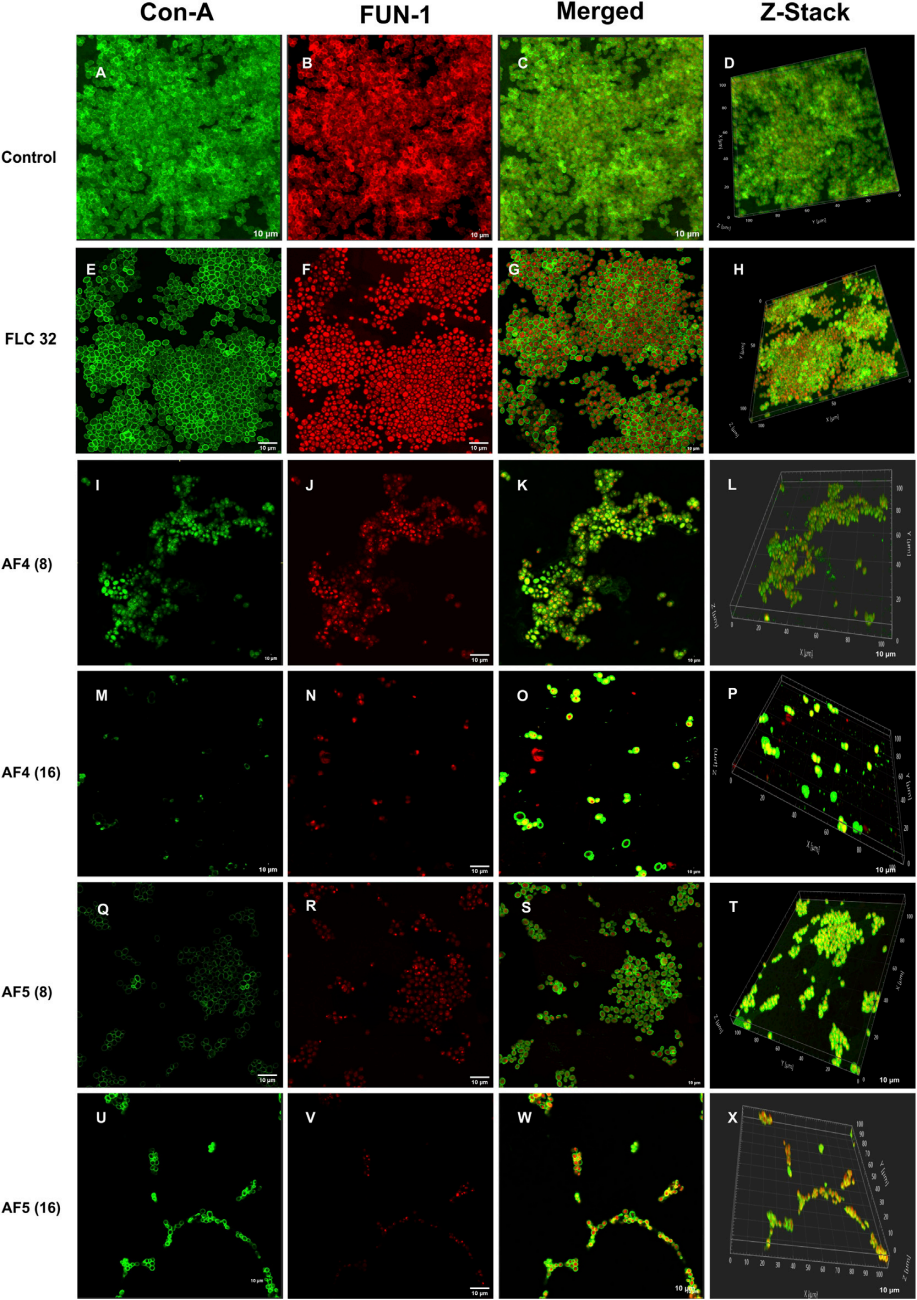
CSLM images of biofilm-associated *CG* cells. Concanavalin A-Alexa 488 (green), and FUN-1 (red) staining at 60 × 2 oil immersion objective and 2× magnification. Con-A stains the extracellular polysaccharides and FUN-1 stains the metabolically active cells. Each image **(A–D)** is control (untreated), and **(E–H)** is treated with FLC 32 μg/mL, and **(I–P)** are treated with AF_4_ 8 and 16 μg/mL respectively. **(Q–X)** AF_5_ 8 and 16 μg/mL respectively. 3D reconstruction images were obtained from Z-stack. Scale bar: 10 µm.

The CSLM was used to examine the effects of combinatorial treatments of novel lipopeptides with FLC at varying concentrations, AF_4_/AF_5_ at two concentrations (8 and 16 μg/mL) and FLC alone on the biofilm architectures of 24 h *C. glabrata* biofilms and compare them with controls. The untreated 24 h biofilms not exposed to the drug displayed densely packed architecture that appeared as a green mat. The noticeable amount of extracellular polymeric materials resulted on account of EPS binding by concanavalin A–Alexa Fluor 488 conjugate, especially in 24 h old biofilm (Figures 3A–D). FLC 32 μg/mL-treated 24 h biofilm showed an umpteen number of metabolically viable cells in thick clusters that metabolized the FUN-1 with reddish fluorescence from inside accompanying the Con A-Alexa Fluor 488 conjugate bound EPS (Figures 3E–H) (Supplementary Figures S5A, B). On the contrary, AF_4_/AF_5_ (8 and 16 μg/mL) exhibited an insignificant number of cells or cell clusters that were mainly either not viable or not embedded in the biofilm (Figures 3I–X) (Supplementary Figures S5C, D).

**FIGURE 4:**
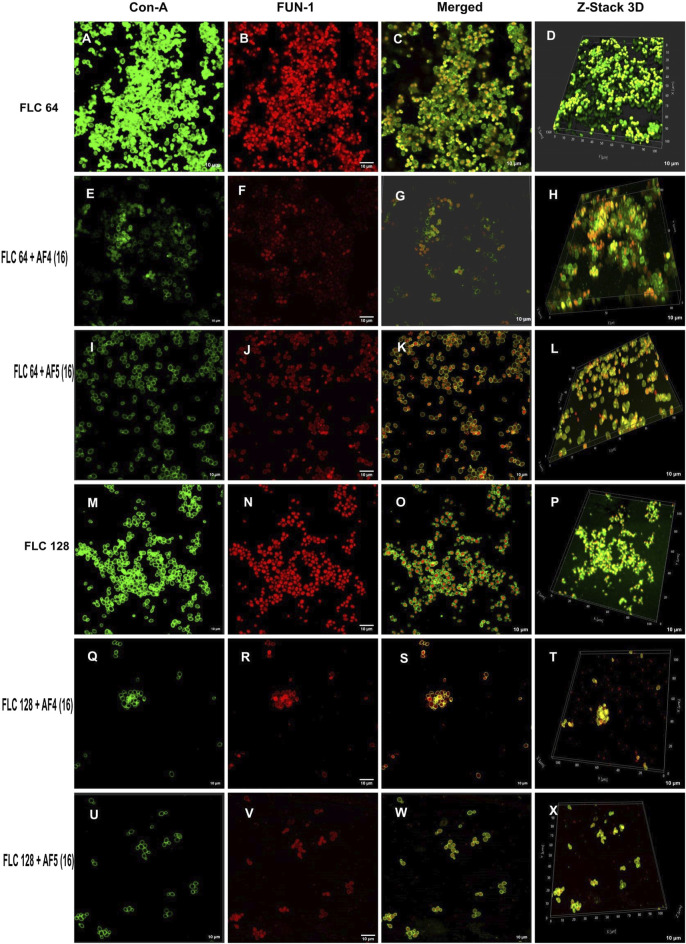
Confocal images of 24 h biofilm of *CG* ATCC 2001 treated with varying concentrations of FLC alone and in combination with AF_4_/AF_5_. Images were acquired using the confocal scanning laser microscope, con-A, Alexa Flour 488 conjugate (green), and FUN-1 (red) staining at ×60 oil immersion objective and ×2 magnification. Con-A stains the extracellular polysaccharides, and FUN-1 stains the metabolically active cells. The images **(A–D)** show biofilms treated with 64 μg/mL, while the images **(M–P)** show biofilms treated with FLC 128 μg/mL. Images **(E–H)** depict biofilms treated with FLC 64 μg/mL and AF_4_ (16 μg/mL), while images **(I–L)** show biofilms treated with FLC 64 μg/mL and AF5 (16 μg/mL). Images **(Q–T)** show biofilms treated with FLC 128 μg/mL and AF_4_ (16 μg/mL), and **(U–X)** show biofilms treated with FLC 128 μg/mL and AF_5_ (16 μg/mL). Z-stack 3D reconstruction images were obtained, and the scale bar is 10 µm.

Morphological and structural features of *C. glabrata* biofilm (Figures 4A–D), when treated with FLC 64 μg/mL alone, appeared similar to those of control groups, with tightly packed structures visible in confocal images. The effect of FLC 128 μg/mL alone (Figures 4M–P) on biofilms was found to be comparable to the control and FLC 64 μg/mL. However, the confocal images (Figures 4E–H) of AF_4_ (16 μg/mL)/FLC 64 µg/mL-treated biofilms showed a mixture of metabolically active and inactive cells, and (Figures 4Q–T) images of AF_4_ (16 μg/mL) and FLC 128 μg/mL treated biofilms mostly exhibited metabolically inactive cells with no detectable biofilms. A stark contrast in fluorescence pattern between untreated 24 h *C. glabrata* biofilm and FLC (64 μg/mL) plus AF_5_ (16 μg/mL)-treated ConA-FUN-1 stained biofilm was observed (Figures 4I–L), which indicates that 24 h biofilm had viable yeast cells as FUN-1 was metabolized, showing orange to red fluorescence. The combined effect of AF_5_ with FLC 128 μg/mL on 24 h grown biofilm was evident, with a single small cluster of damaged cells reflecting only partial eradication of biofilms after 48 h (Figures 4U–X).

### 3.6 COMSTAT analyses

**FIGURE 5:**
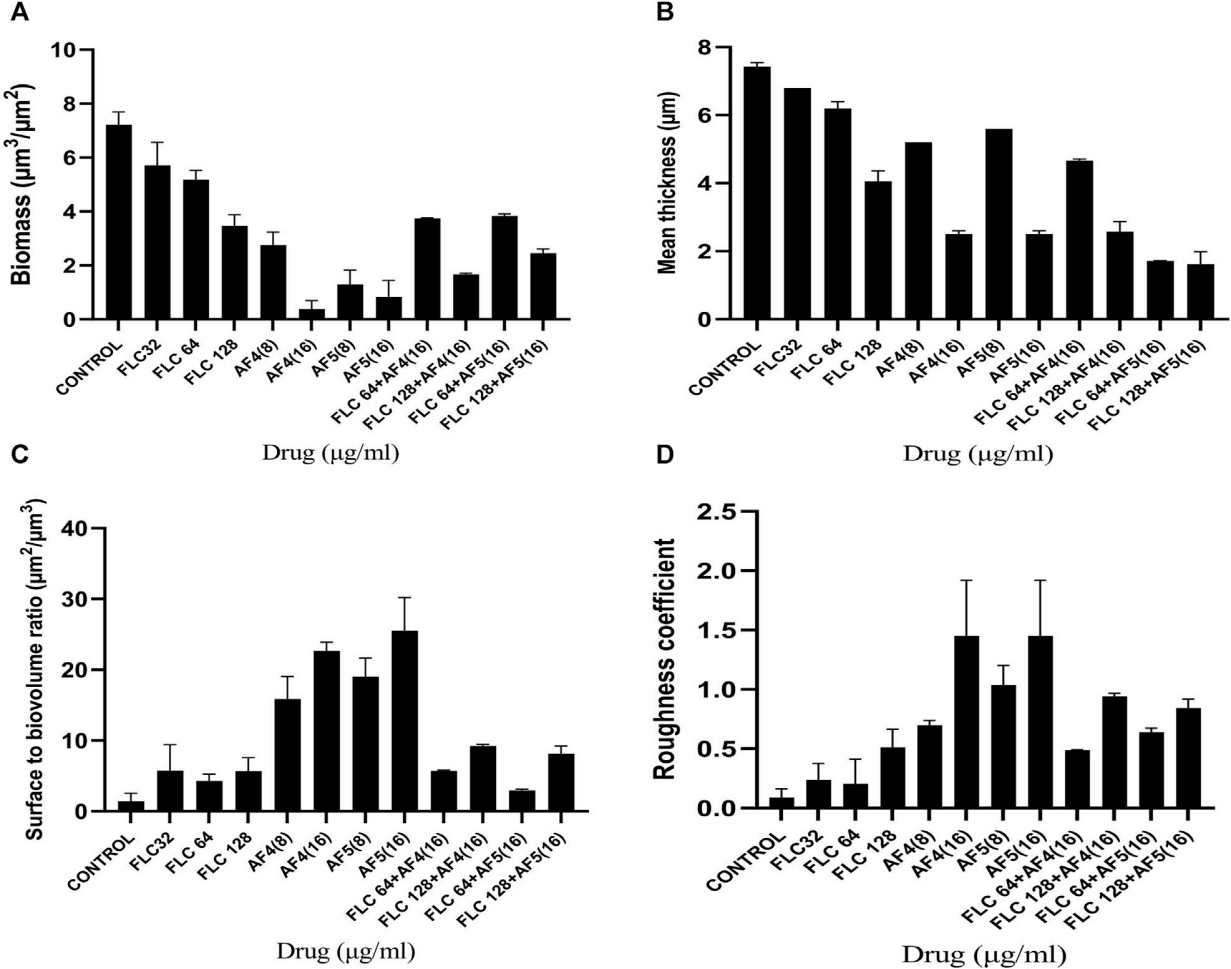
CSLM-COMSTAT analysis of various parameters of the *CG* 2001 biofilms untreated and treated at 24 h. **(A)** Biomass (µm^3^/µm^2^), **(B)** Mean thickness (µm), **(C)** Surface to bio-volume ration (µm^3^/µm^2^), and **(D)** Roughness coefficient.

The reduction in biomass and maximum thickness in *C. glabrata* biofilm were assessed using COMSTAT software. The analysis showed that the combinations of AF_4_/AF_5_ (16 μg/mL) and FLC 128 μg/mL treated biofilm exerted a decrease in total biomass and mean thickness when compared to different concentrations and control (untreated) (Figures 5A, B), (Supplementary Table S1). The AF_5_ (16 μg/mL) combination with FLC 128 μg/mL showed the least average biofilm thickness. The roughness co-efficiency was highest in AF_4_ (16 μg/mL) plus FLC 128 μg/mL followed by AF_5_ (16 μg/mL) plus FLC 128 μg/mL, and a notable increment was found in the surface to bio-volume ratio with treatment of AF_4_ and AF_5_ (16 μg/mL) (Figures 5C, D).

### 3.7 Determination of intracellular ROS generation

**FIGURE 6:**
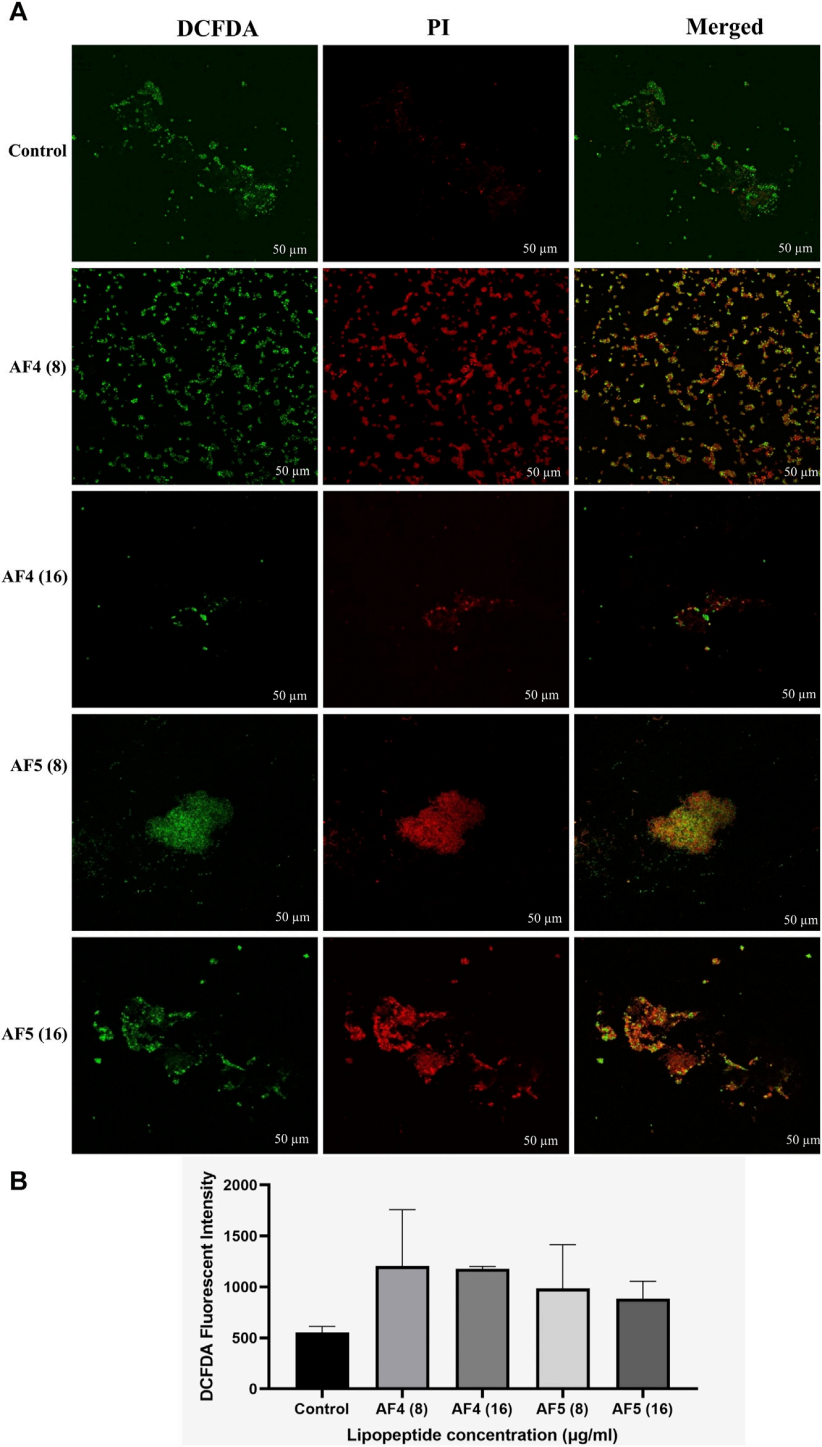
**(A)** Intracellular ROS generation in *C. glabrata* pre-formed biofilm. The CSLM images of *C. glabrata* biofilm cells stained with DCFDA and PI after antifungal lipopeptide treatment. The units of the values in brackets and FLC are µg/mL. Scale bar 50 μm. **(B)** The ROS generation in AF_4_ and AF_5_ treated biofilm were measured using DCFDA dye in terms of fluorescence intensity at excitation of 485 nm and emission of 520 nm. Data represent the means ± SD of two individually performed experiments.

The fluorescent DCFDA was used to measure the intracellular ROS generation in *C. glabrata* biofilms treated with AF_4_/AF_5_ at 8 and 16 μg/mL. The intensities of DCFDA and PI in AF_4_/AF_5_-treated *C. glabrata* biofilm cells were visualized by CSLM, where green emission shows ROS generation and red emission indicates cell membrane damages (Figures 6A, B). The level of intracellular ROS accumulation was higher in the AF_4_ (8 μg/mL)-treated biofilm cells, when compared to the 16 μg/mL treatments due to the higher number of cells, which is correlated to CSLM data (Figure 6B). The DCFDA fluorescent intensities (Figures 6A, B) of control samples revealed the fluorescent intensity was lower than that of lipopeptide-treated samples AF_4_ (8 and 16 μg/mL) and AF_5_ (8 and 16 μg/mL). PI is a DNA-binding fluorescent dye that is unable to penetrate healthy cell membranes. The data suggest lipopeptide-mediated ROS generation results in biofilm cell damage at both concentrations of lipopeptides but is almost equivalent to DCFDA-fluorescent intensities at 8 and 16 μg/ml as the number of cells present in treated biofilms was less in 8 μg/mL-treated biofilms as compared to 16 µg/mL-treated biofilms. CLSM images generated from AF_4_/AF_5_ treatments showed the fluorescence in red and green, which indicate dead and live cells, respectively. The cell-damaging effect driven by the accumulation of ROS was demonstrated by the binding ability of PI to the DNA of damaged cells. The fluorescence channel of bound PI was observed to be higher in cells exposed to AF_4_/AF_5_ treatment than in untreated cells (Figure 6A).

### 3.8 FTIR spectroscopy analyses of treated biofilms

**FIGURE 7:**
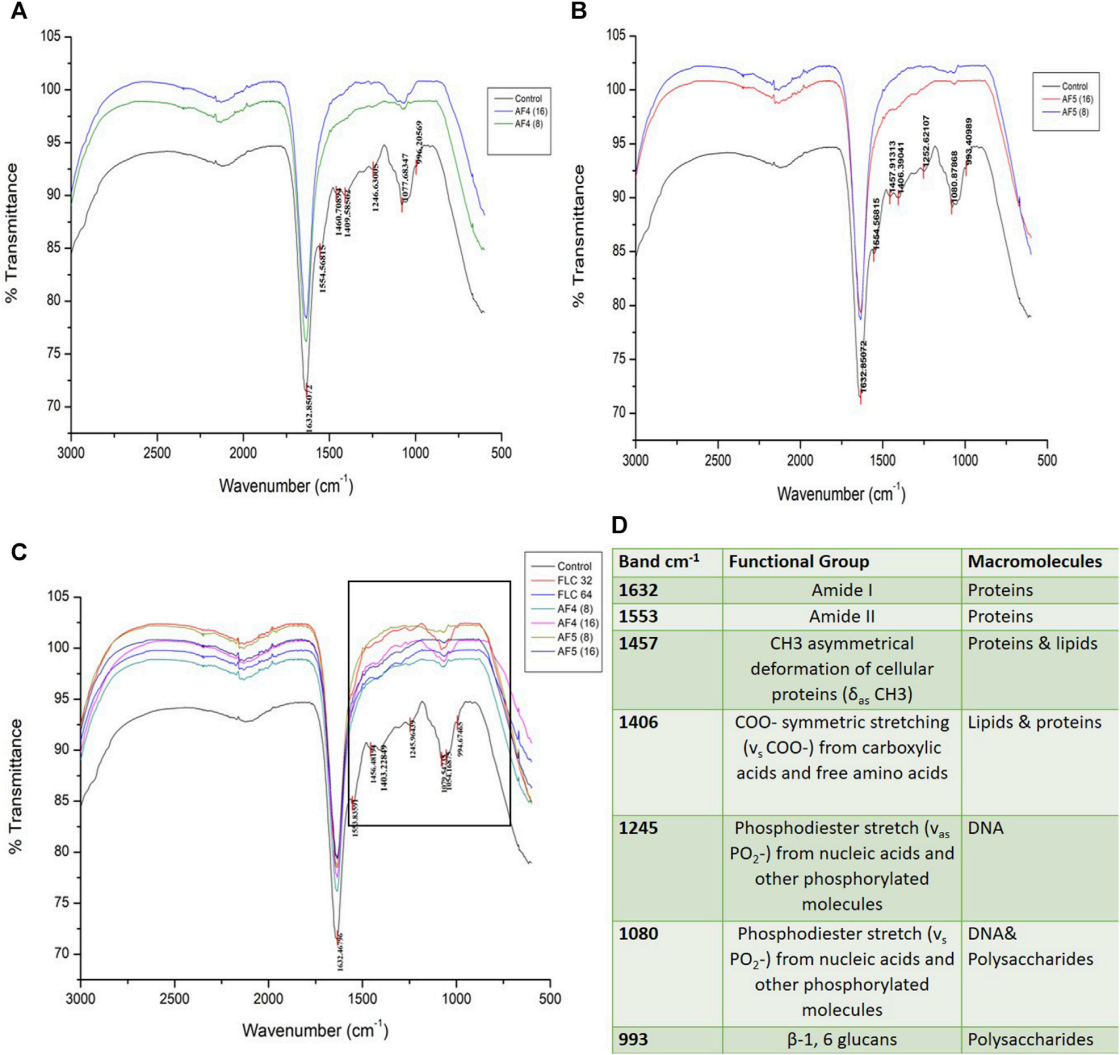
Attenuated total reflectance Fourier transform infrared (ATR-FTIR) spectra analysis of untreated *C. glabrata* 24 h biofilms **(A,B)**, AF_4_ and AF_5_ (8 μg/mL and 16 μg/mL) treated biofilms respectively, **(C)** shows spectra of merged AF_4_ and AF_5_ along with FLC (32 and 64 μg/mL)-treated biofilms, **(D)** Main absorption bands and assignments for ATR-FTIR spectra of *C. glabrata* biofilms.

The ATR-FTIR was used to analyse the biochemical changes induced by the antifungal lipopeptides on 24 h formed *C. glabrata* biofilm. The spectral region ∼3,000–500 cm^−1^ was examined, which includes spectral regions where chemical species indicate the significant components of biofilms, e.g., proteins, lipids and polysaccharides. FTIR spectra of the biofilms revealed the variation of the characteristic absorbance profiles of the *C. glabrata* biofilm functional groups in the 1700–900 cm^−1^ spectral range. The FTIR spectra of *C. glabrata* biofilm functional groups (Figures 7C, D) showed features similar to those previous reports (Nithyanand et al., 2015; Pebotuwa et al., 2020; Villa et al., 2021). The most prevalent signals in these spectra originated from functional groups of amide and lipids and polysaccharides. The observed results displayed the spectra of following peaks; amide I (1,632 cm^−1^), and amide II (1,553 cm^−1^), COO^−^ symmetric stretching of carboxylic acid (1,457 cm^−1^, 1,406 cm^−1^) of lipids and proteins, phosphodiester stretch (1,245 cm^−1^, 1,080 cm^−1^), and β-1,6 glucans (993 cm^−1^) polysaccharides. However, FLC (64 μg/mL) also shows significant effect on the attenuating corresponding peaks related to biofilm components (Figure 7C). Overall, the significant effect of AF_4_/AF_5_ lipopeptides on *C. glabrata* 24 h biofilms were revealed by the alteration of intensity of ATR-FTIR fingerprint from the range of (1700–900 cm^−1^) with and without lipopeptide-treatments as shown in the (Figures 7A, B).

### 3.9 Estimation of exopolysaccharides (EPS) of treated biofilms

**FIGURE 8:**
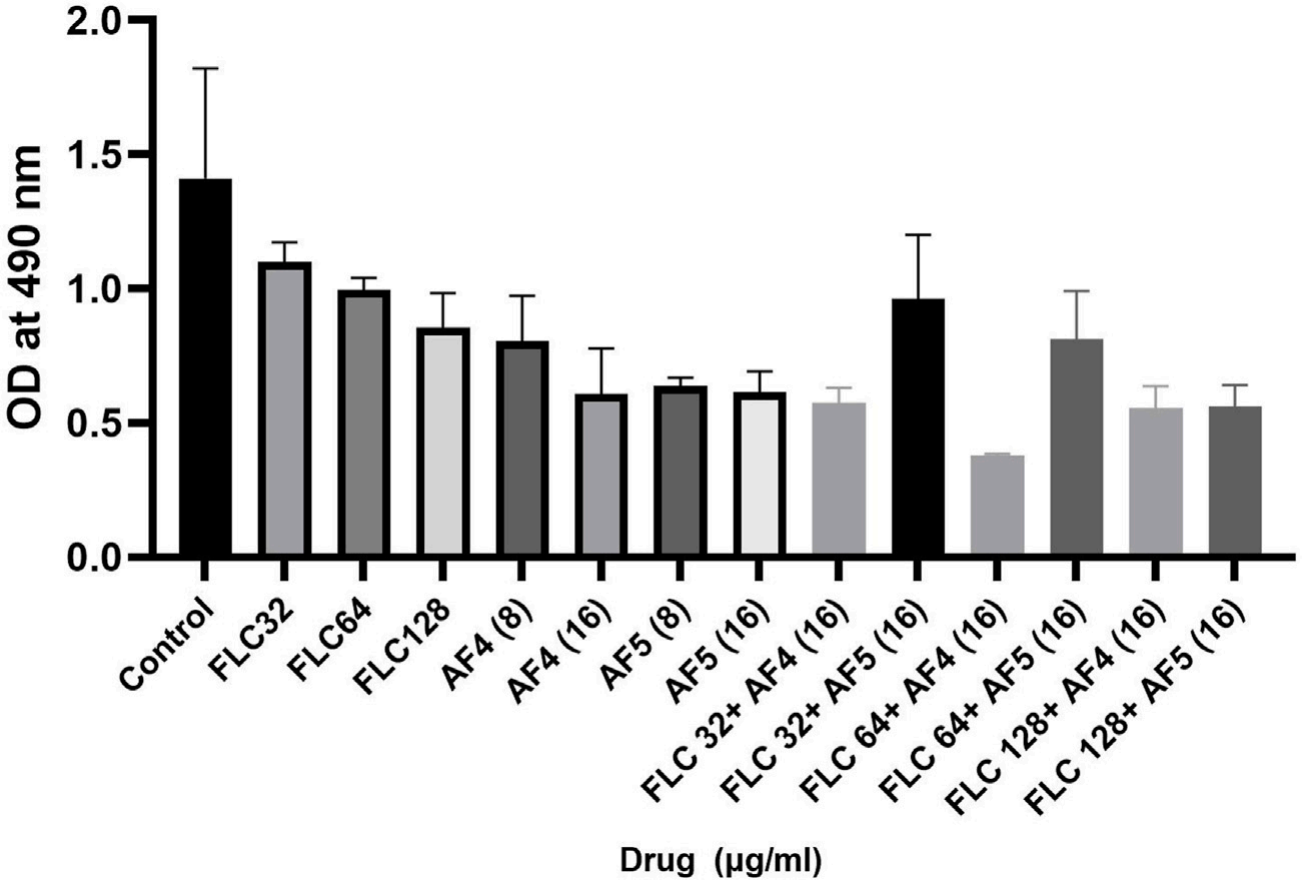
Effects of FLC, AF_4_/AF_5_ alone and various combinations of AF_4_/AF_5_ and FLC at different concentrations on the EPS layer of *CG* biofilms. AF_4_ at 16 μg/mL, and AF_5_ at 8 μg/mL and 16 μg/mL showed reduction in exopolysaccharide.

Fungal cell wall polysaccharides are also important constituents of the *Candida* biofilm exopolymeric materials (Chandra et al., 2001; Kuhn et al., 2002). The phenol sulfuric assays were performed to examine whether AF_4_/AF_5_ alone, fluconazole and AF_4_/AF_5_ combinations had the ability to diminish the EPS layer formed by *C. glabrata* biofilms. Compared to the control group, treatment with AF_4_/AF_5_ at a concentration of 16 μg/mL resulted in a decreased EPS (extracellular polymeric substances) content, as shown in (Figure 8). Similarly, FLC 64/AF_4_ (16 μg/mL), FLC 128/AF_4_ (16 μg/mL), and FLC 128/AF_5_ (16 μg/mL) treatments also showed significant reductions in EPS content compared to the control group.

## 4 Discussion

Biofilms produced by NCAC species, such as *C. tropicalis*, *C. parapsilosis* and *C. glabrata*, exhibited reduced antifungal susceptibility by restricting the penetration of antifungal through the ECM (Hawser and Douglas, 1994; Silva et al., 2012). Biofilm-mediated drug resistance are associated with high cell density, alterations in cellular metabolism, cell signaling, quorum sensing, and presence of persister cells (Ramage et al., 2012; Taff et al., 2013). The combination of antimicrobial resistance and biofilm formation leads to infections that appear to be unmanageable (Campoccia et al., 2006; Martinez et al., 2019). The antifungal drug resistance in *C. glabrata* involves overexpression or upregulation of genes such as ATP-binding cassette (ABC) transporter genes like CgCDR1, CgCDR2, and ergosterol synthesis pathway enzymes (Thompson et al., 2008; Yoo et al., 2010). The efflux of azole drugs are reported to be facilitated by ABC transporters and major facilitator (MF) transporters superfamily efflux pumps (Coleman and Mylonakis, 2009). Other than fluconazole, *Candida* species also exhibit resistance to azoles such as isavuconazole, clotrimazole, itraconazole, ketoconazole, and the increased rate of multidrug resistance (MDR) is an alarming concern found in clinical isolates from patients in pathophysiological states, as reported by (Tsega and Mekonnen, 2019). The resistance to echinocandins in *C. glabrata* is associated with specific mutations in the Fks1p and Fks2p genes, which encode β-1,3 glucan, an essential component of the cell wall (Katiyar et al., 2012; Coste et al., 2020). *C. glabrata* pathogenicity is associated with various virulence factors, such as adhesion to the human epithelial surface, secreting enzymes, and biofilm formation (Hassan et al., 2021). The biofilm formation by this yeast species enables the yeast cells to develop antifungal resistance at high drug concentrations. Reports are not scanty to indicate that antifungals are less effective against biofilm-producing organisms, and the development of new antifungal agents for the treatment of recalcitrant biofilm-driven infections is therefore of interest, and the present study was attempted to address this concern The combination of two antimycotics of different natures could be a strategy to prevent the development of drug resistance and overwhelm biofilm resistance. Several studies indicate the likelihood of synergistic success due to the different mechanisms of action of two cadres of compounds such as various antimicrobial peptides (AMPs) and azoles (Nett et al., 2011; Mora-Navarro et al., 2015; Czechowicz et al., 2021). Appropriate combinations of antifungal agents may be helpful to overcome drug resistance; in this light, exploring the synergistic or additive effect of lipopeptides with other existing antibiotics may hold the future key (Patel et al., 2015). The potency and low cytotoxicity of newfangled compounds may decimate the overall use of antibiotics, and the development and spreading of antimicrobial resistance may be plummeted by using antimicrobial lipopeptides or appropriate AMPs, either alone or in combination with low doses of antibiotics (Claeys et al., 2014; Wiman et al., 2023). Two ultrashort cationic cyclic lipopeptides (USCLs), as previously reported (Neubauer et al., 2020), showed antibiofilm activities, wherein the mean biofilm inhibitory concentrations were 87 μg/mL (Neubauer et al., 2020). Very recently, two USCLs were reported with a minimum biofilm eradication concentration of 64 μg/mL against isolates from vulvovaginal candidiasis (Neubauer et al., 2020; Czechowicz et al., 2021). A battery of natural and synthetic peptides has been reported to suppress the biofilm’s metabolic activity (Nieminen et al., 2014; Nithyanand et al., 2015). AF_4_ and AF_5_ lipopeptides at two different concentrations alone have exhibited unarguably *in vitro* anti-biofilm efficacy comparable to or higher than these compounds in inhibiting the biofilm metabolism. According to a report, the *B. subtilis* AC7 lipopeptide at a very high concentration (2000 μg/mL) reduced biofilm formation by up to 57% in *C. albicans* (Ceresa et al., 2016). *C*. *glabrata* uses adhesion and biofilm formation to better adapt to the environment and infect the host (Weerasekera et al., 2016; Hosida et al., 2018). It is evident from our results that untreated biofilms grew continuously and reached a mature phase within 24 h in RPMI-1640. In contrast, AF_4_/AF_5_-treated and FLC and AF_4_/AF_5_- combination-treated biofilms exhibited limited growth and showed dismantling of pre-formed biofilm. An observation of this study was the significant metabolic activity of *Candida* cells in the early-adhesion phase by 6 h, as revealed by the XTT reduction assays (Figure 1B). Compared to young and mature *Candida* biofilms of 24 h, the early phase displays a relatively lower cell density. *C*. *glabrata* ATCC 2001 exhibited good growth (Figure 1A), (Supplementary Figure S5A) at 24 h, as revealed by the significant biomass and metabolic activity (Figure 1B).

A separate study conducted previously showed that FLC at only very high concentrations (625 and 1,250 μg/mL) showed a significant reduction in 24 h *C. glabrata* ATCC 2001 biofilm biomass (Fonseca et al., 2014). A separate group (Mota et al., 2015), reported the high biofilm-inhibitory concentration (312.5 μg/mL) of FLC against 24 h pre-formed *C. glabrata* ATCC 2001. When the findings of the previous studies (Fonseca et al., 2014; Mota et al., 2015) are taken together, where 312–1,250 μg/mL of FLC were used to reduce or inhibit the biofilm formation, the present study has shown that using 16 μg/mL AF_4_/AF_5_ and 64 μg/mL FLC can nearly disrupt the mature biofilms. In another study, higher biomass formation by susceptible *C. glabrata* (*CgS*) in the presence of FLC when compared to *CgS* in the absence of FLC was reported (Panariello et al., 2018). Different microscopy techniques, such as SEM and CLSM, coupled with the modelling software COMSTAT, were employed to analyse the biofilm structures and architecture parameters (Seneviratne et al., 2009). The control (untreated) produced a well-organized biofilm that grew as densely packed structure as demonstrated by scanning electron micrographs.

The mechanistic insight gained for the biofilm microenvironments is enabling the development of targeted therapeutic strategies to prevent biofilm formation and combat preformed biofilms (Oshiro et al., 2019). Several studies emphasize the need to explore the possibility of reaping the beneficial effects of combinations of conventional antimycotics such as FLC with various AMPs*/*AFPs against *Candida*-associated biofilms, because of the different mechanisms of action offered by two different classes of compounds (Mora-Navarro et al., 2015; Suchodolski et al., 2020). Antimicrobial peptides have been deemed to be highly promising substitutes to treat biofilm-embedded *Candida* cells (Batoni et al., 2011). Most of the currently used antifungal agents have a specific mechanism of action. For instance, azoles act by interfering with the biosynthesis of membrane ergosterol. In contrast, AMPs exert their antimicrobial activity by binding to the cellular membrane and then increasing its permeability. Also, due to the conspicuous mechanism of action of AMPs, organisms are less prone to developing drug resistance (Onyewu et al., 2003). It may be speculated that, since AF_4_/AF_5_ might have possibly induced *Candida* cell membrane permeabilization, the FLC concentration that is required to reach the cytoplasm and subsequently inhibit ergosterol biosynthesis could be decreased in the presence of the lipopeptide. It may be concluded that AF_4_ and AF_5_ lipopeptides may hold greater promise as potential treatment options for these clinical isolates.

Research carried out to date suggests a positive correlation between the ability to inhibit or eradicate biofilms and the length of the fatty acyl chain. Since the AF_4_/AF_5_ lipopeptides have a long fatty acid chain (Ramachandran, et al., 2018), it may be hypothesized that by virtue of having a long fatty acid moiety, the lipopeptide acquires the potential to inhibit or nearly dismantle the preformed biofilm more efficiently, regardless of the nature of their respective component (Paluch et al., 2021). The fatty acid moieties present in AF_4_/AF_5_ might trigger the induction of oxidative stress and ROS generation in the *Candida* cells, and therefore treated *Candida* biofilms showed a significant alterations in the levels of ROS, as evident from DCFDA fluorescence emission. The data (Figure 6B) suggest considerable ROS generation in the pre-formed biofilm of *C. glabrata* at a lower concentration of the lipopeptide (8 μg/mL) as compared to the control, while at as higher concentration (16 μg/mL) the fluorescent intensity was found to be less (Figure 6B) as the number of cells in the biofilm decreased significantly to generate the ROS. Additionally, fluorescence absorbance of PI showed significant cell permeabilization as a result of cell membrane damages in the *C. glabrata* biofilms due to oxidative damage induced by the lipopeptides (Figure 6A). The disruptions of biofilms observed in the present above-mentioned studies have been supported by the alterations in carbohydrate, DNA, and protein profiles in the AF_4_/AF_5_-treated biofilms by FTIR analyses revealing the biofilm disruption. The very negligible intensity of the amide II (1,553 cm^−1^) band in AF_4_/AF_5_-treated biofilms which is otherwise considered as a marker of biofilm biomass provides the evidence of the significant effect on biofilm as compared to the control (untreated). Biosurfactant properties tend to destabilise the structure and permeability of membranes and appear to modify the surface properties of the substratum, negatively affecting cell viability and adhesion, restricting biofilm formation, or enabling partial disruption of preformed biofilms (Fracchia et al., 2015; K. Satputea et al., 2016). Therefore, prior application of these lipopeptides to medical devices may be deemed a preventive strategy to delay the onset of pathogenic biofilm growth.

## 5 Conclusion

This study investigated the effectiveness of two novel antifungal lipopeptides, in preventing biofilm formation by *C. glabrata* 2001 and other clinical isolates. The present study has uncovered robust antibiofilm properties of two lipopeptides highlighting a synergistic impact when combined with FLC. The combination of AF_4_ and AF_5_ with FLC has proven highly effective in reducing biofilm development and dismantling established biofilms as well. The results obtained pave the way for further exploration, utilizing these compounds as promising candidates for combating *Candida* biofilms. Their roles as anti-biofilm agents present a valuable opportunity to improve disinfectant solutions and optimize surfaces, especially in the domain of medical devices like catheters. Ultimately, this investigation underscores the substantial potential of these lipopeptides as leads in addressing infections associated with *Candida* biofilms.

## Data Availability

The original contributions presented in the study are included in the article/Supplementary Material, further inquiries can be directed to the corresponding author.
