# Supplementary material for: Two promising Bacillus-derived antifungal lipopeptide leads AF4 and AF5 and their combined effect with fluconazole on the in vitro Candida glabrata biofilms
Source: Front Pharmacol. 2024 Apr 19;15:1334419. doi: 10.3389/fphar.2024.1334419 (PMC11066293; doi:10.3389/fphar.2024.1334419)
Supplement: Supplementary file 1 [file DataSheet1.PDF]

The Study of Promising *Bacillus*-derived Antifungal Lipopeptide Homologues AF_4/AF_5 and Its Combined Effect with Fluconazole on the *In vitro Candida glabrata* Biofilm

Madduri Madhuri, Shivaprakash M. Rudramuruthy , Utpal Roy

Suppl. Figure.1

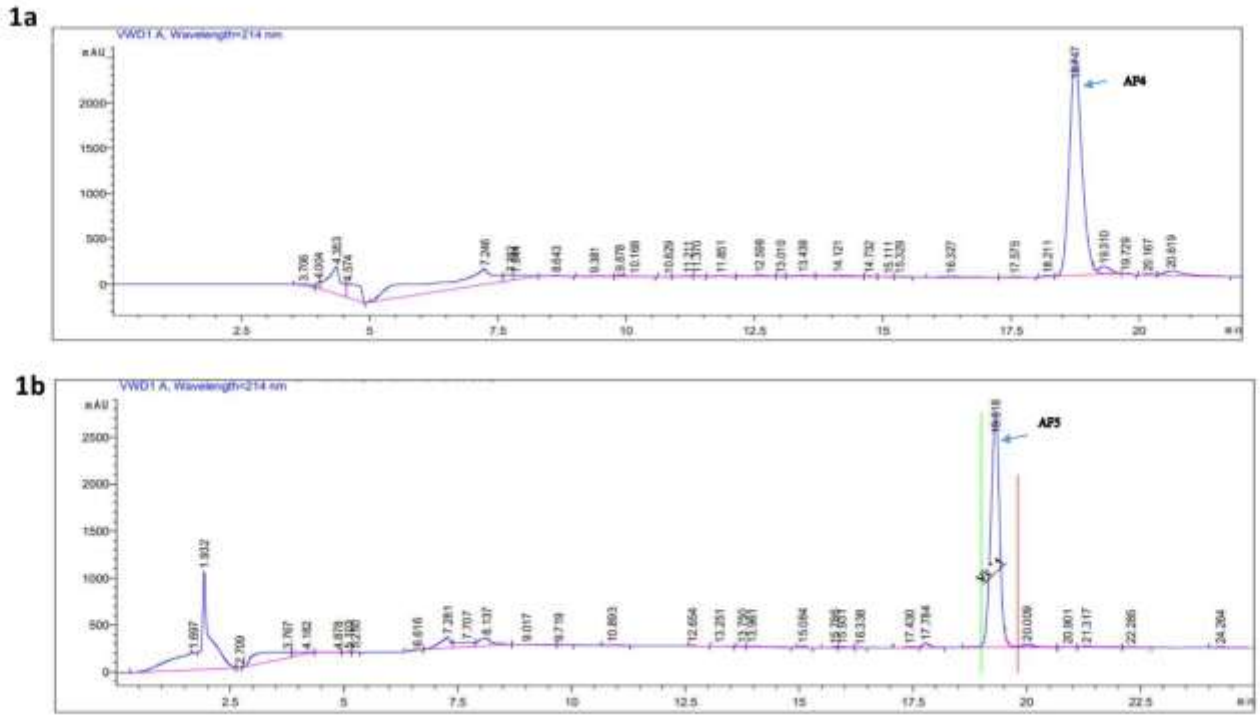

Fig. 1a, b. RP-HPLC profiles at the analytical scale. The chromatograms of AF_4 (1a) and AF_5 (1b) are shown here.

Suppl. Figure. 2.

a.

b.

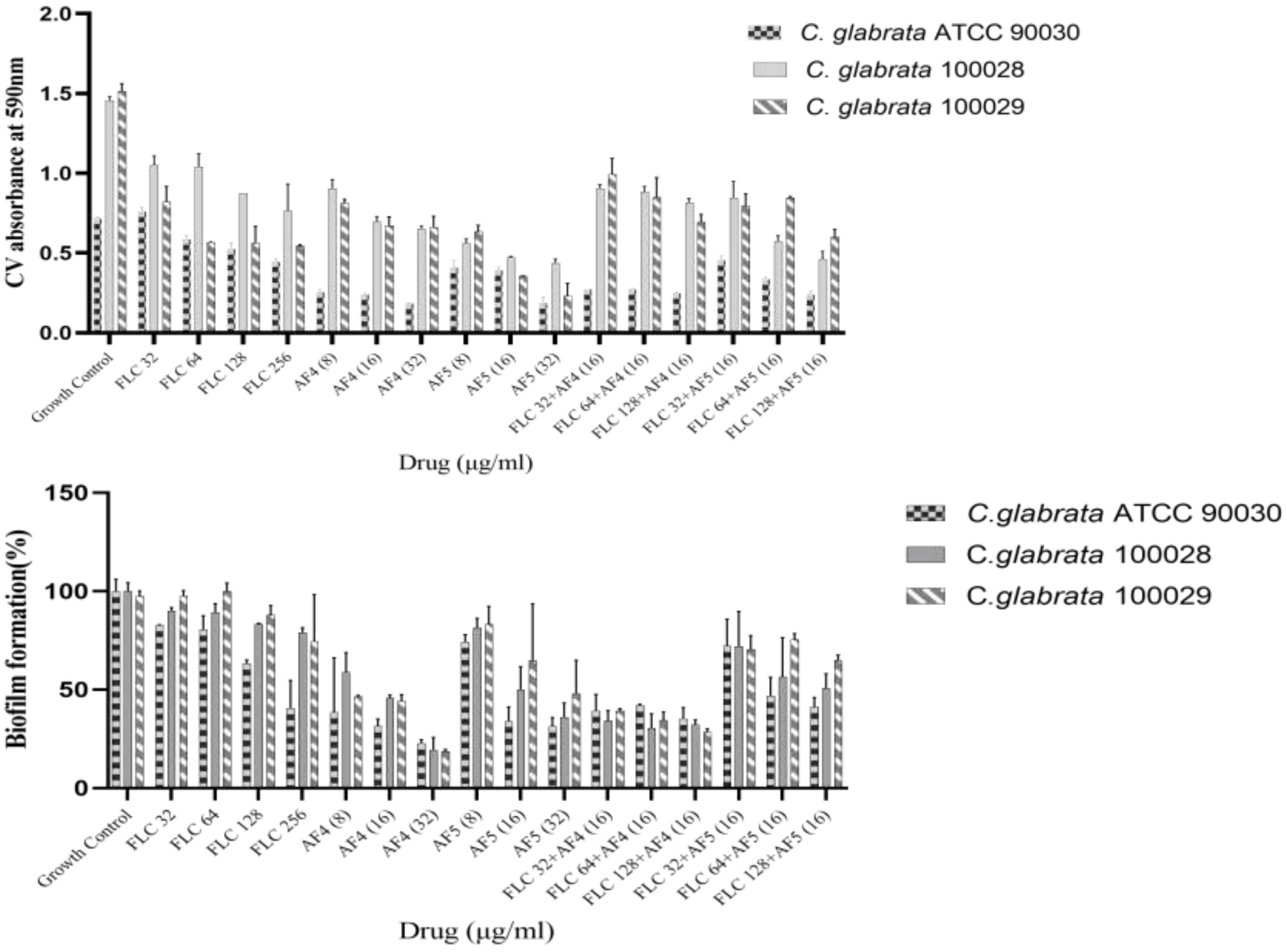

Suppl. Figure. 2 Effect of FLC alone and FLC plus (AF_4/AF_5) on *C. glabrata* ATCC 90030, and two clinical isolates NCCPF 100028 and 100029 on 24h pre-formed biofilm. **2a.** Mean absorbance values at 590 nm obtained from the total biomass quantification by CV assay **2b.** Results were normalized to control (untreated), which was taken as (100%). All values are means plus standard deviations.

Suppl. Figure. 3.

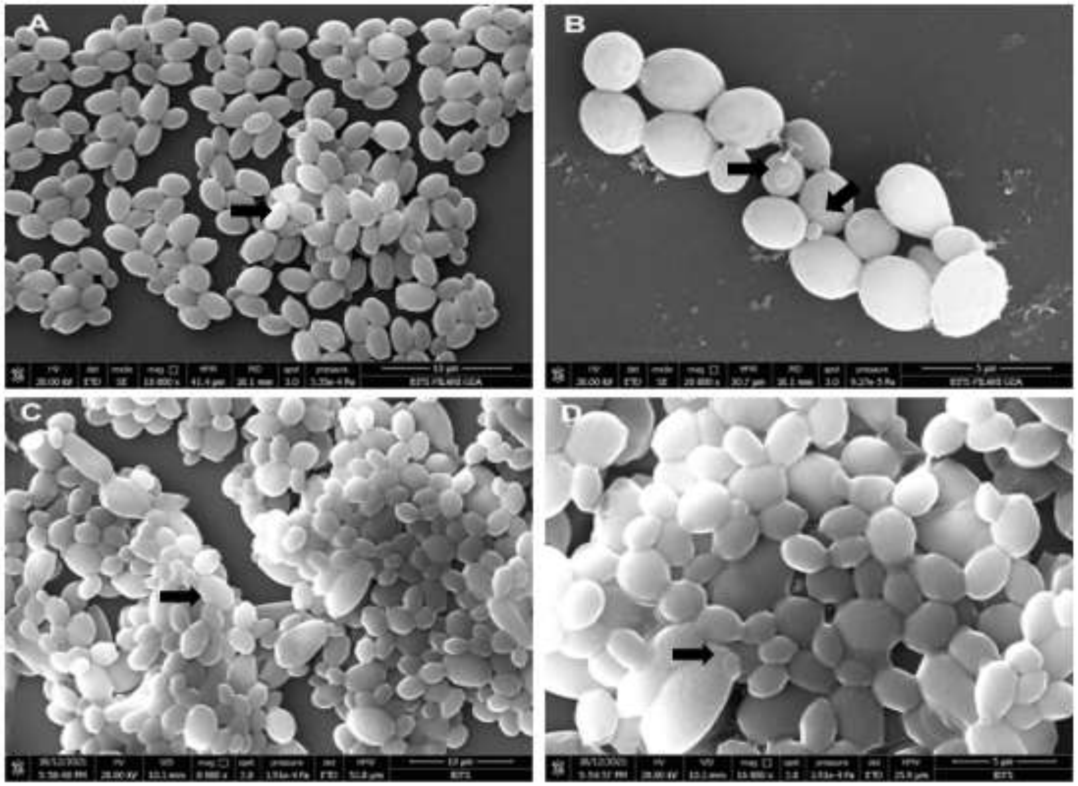

Suppl. Figure. 3. Scanning electron micrographs of *CG* 2001 biofilm. (A-B) developmental-stage (6h) biofilm control (untreated) and (C-D) 24 h preformed biofilm control (untreated) with multi-layered yeast cells. Images are shown in 10 µm and 5 µm scale bars.

Suppl. Figure. 4.

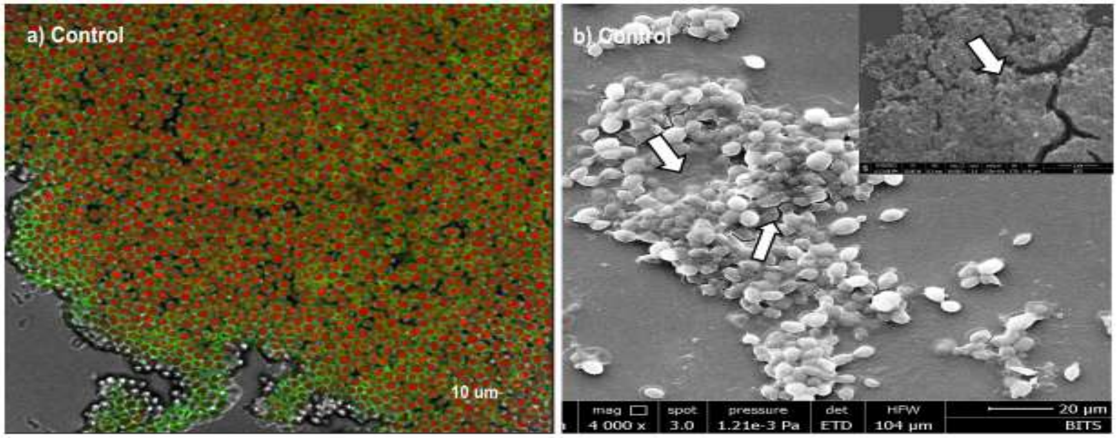

Suppl. Figure. 4a. The CSLM image of 24h biofilm stained by Con-A 488 conjugate and FUN-1 reveals thick biofilm formation along with EPS production. **Fig.4b** The SEM image of untreated (control) *CG* 2001 24h thick biofilm with multi-layered yeast cells covering the surface. Scale bars of SEM and confocal images are 20 and 10 µm respectively.

Suppl. Figure. 5.

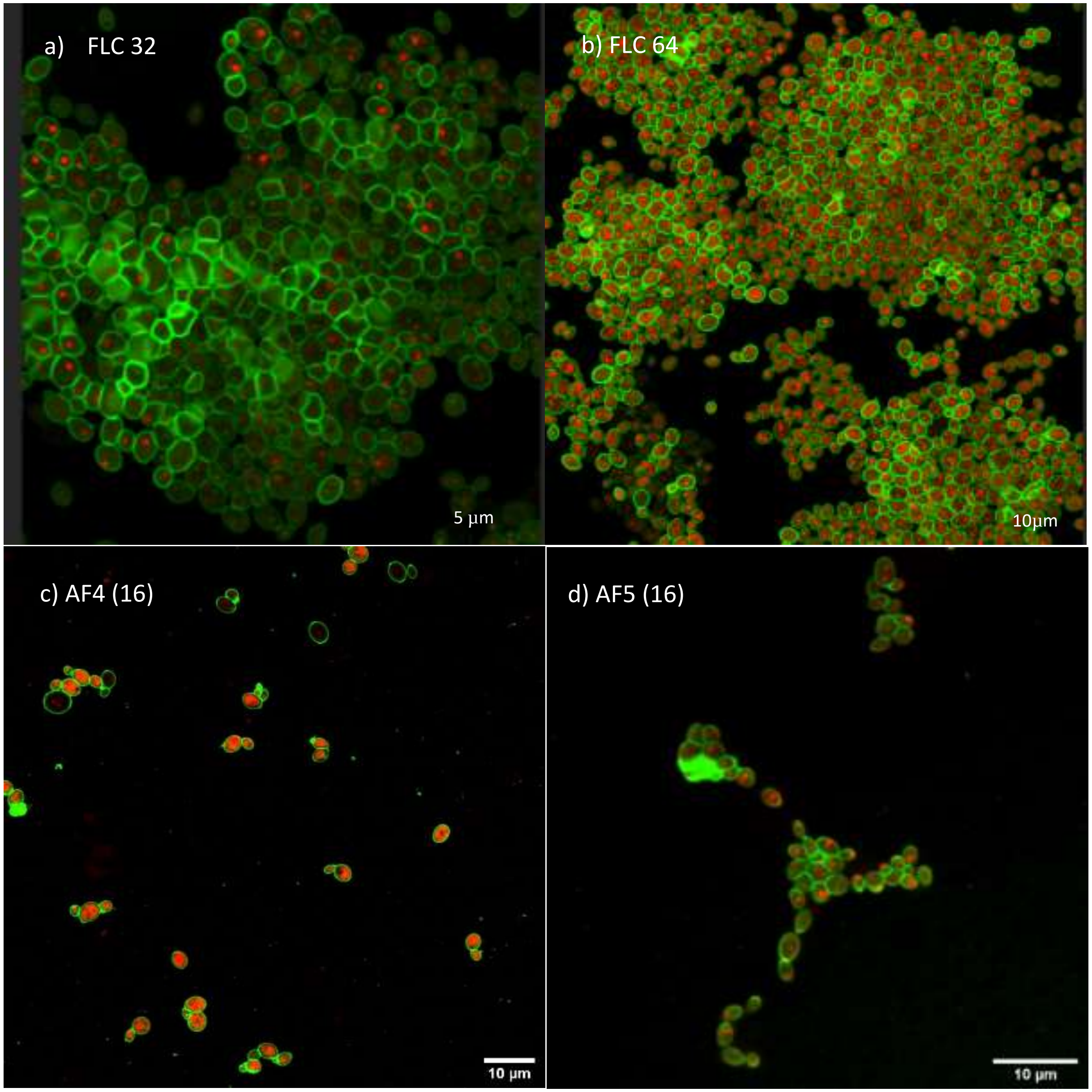

Suppl. Figure. 5. (a, b) the CSLM image of 24h biofilm treated with FLC32 µg/ml stained with Con-A and FUN-1 found thick biofilm formation along with EPS production. **(c, d)** 24 h pre-formed biofilm treated with AF_4 and AF_5 (16 µg/ml) respectively. Scale bars of SEM and confocal images are 10µm.

Suppl. Fig. 6.

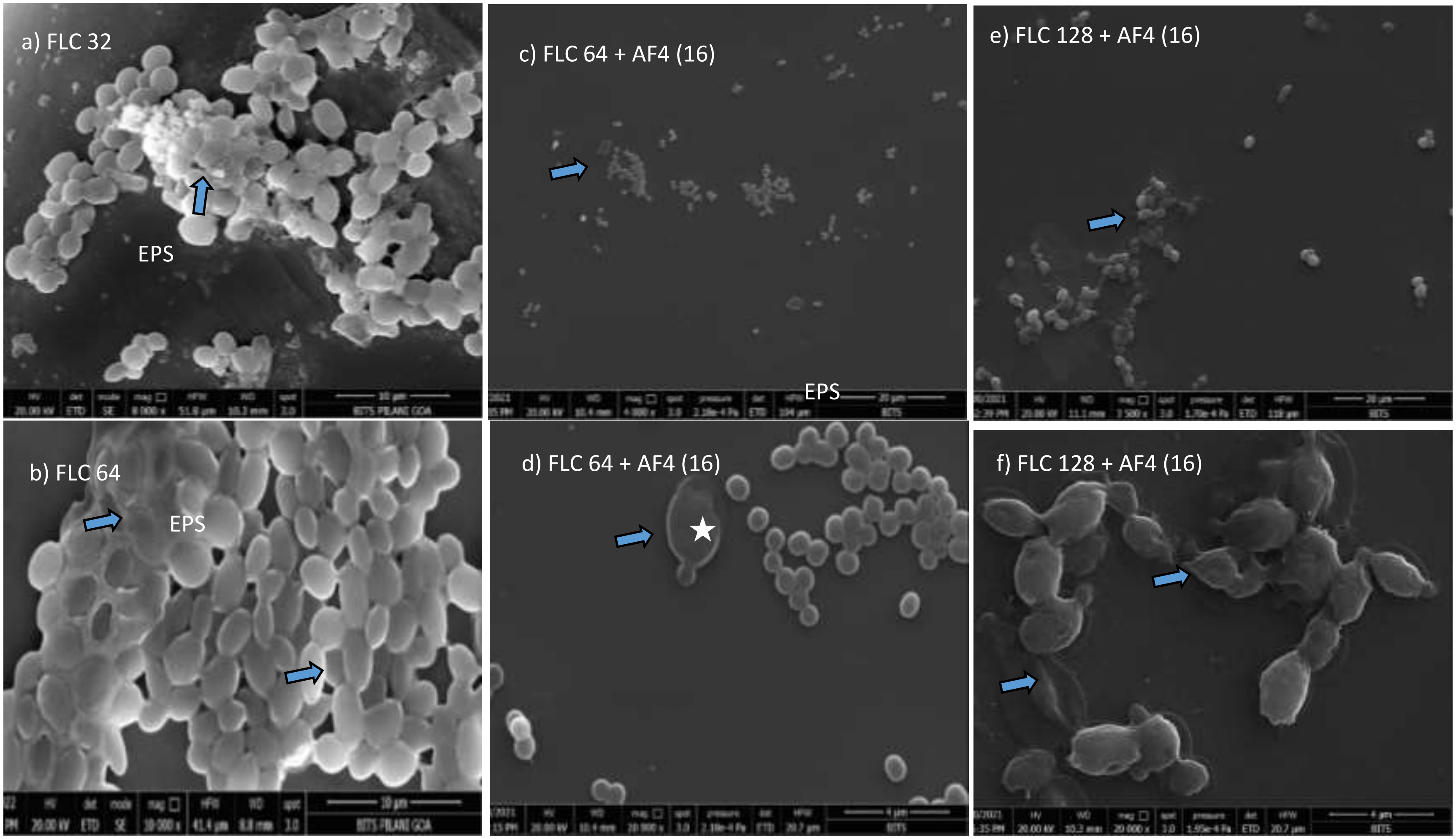

a) FLC 32
EPS
b) FLC 64
EPS
c) FLC 64 + AF4 (16)
EPS
d) FLC 64 + AF4 (16)
★
e) FLC 128 + AF4 (16)
f) FLC 128 + AF4 (16)

Suppl. Figure. 6 (a, b) The SEM images of 24h biofilm treated with FLC 32 µg/ml and FLC 64 µg/ml showing thick biofilm formation along with EPS production. **(c, d)** 24 h pre-formed biofilm treated with AF_4 (16 µg/ml) combination with FLC 64 µg/ml at different scales bars 20 µm and 4 µm. **(e, f)** AF_4 (16 µg/ml) combination with FLC 128 µg/ml at different scales and magnifications. Scale bars 20 µm and 4 µm respectively. Blue arrows indicate cell deformities and asterisk marks indicate specific cell damaged.

Suppl. Table 1. COMSTAT quantitative analysis of *CG* 2001 24 h biofilm parameters of biofilm biomass, average thickness, surface to biovolume ratio and roughness coefficient of treated and control (untreated) biofilms.

**Treatments**	**Biomass ($\mu m^3/\mu m^2$)**	**Surface to biovolume ratio ($\mu m^2/\mu m^3$)**	**Roughness coefficient**	**Local thickness (µm)**
Control	7.2	1.41	0.08	7.42
FLC 32 µg/ml	5.7	5.1	0.08	6.8
FLC 64 µg/ml	5.1	4.28	0.20	6.19
FLC 128 µg/ml	3.4	5.68	0.51	4.05
AF4 8 µg/ml	2.7	15.8	0.69	5.2
AF4 16 µg/ml	0.4	22.6	1.5	2.6
AF5 8 µg/ml	1.3	19.0	1.01	4.6
AF5 16 µg/ml	0.9	22.5	1.33	5.2
FLC 64+AF_4 16 µg/ml	3.7	5.71	0.48	4.66
FLC 128+AF_4 16 µg/ml	1.6	9.24	0.93	2.57
FLC 64+AF_5 16 µg/ml	3.8	2.93	0.63	1.71
FLC 128+AF_5 16 µg/ml	2.4	8.15	0.84	1.61
